# Ionic Conductive Organohydrogel With Ultrastretchability, Self-Healable and Freezing-Tolerant Properties for Wearable Strain Sensor

**DOI:** 10.3389/fchem.2021.758844

**Published:** 2021-10-18

**Authors:** Feng Ji, Min Jiang, Qingyu Yu, Xuefang Hao, Yan Zhang, Junqiu Zhu, Shuiyuan Luo, Junjie Li

**Affiliations:** ^1^ College of Chemical Engineering and Materials Science, Quanzhou Normal University, Quanzhou, China; ^2^ School of Chemical Engineering and Technology, Tianjin University, Tianjin, China; ^3^ Inner Mongolia Key Laboratory of Carbon Nanomaterials, College of Chemistry and Materials Science, Nano Innovation Institute, Inner Mongolia University for Nationalities, Tongliao, China; ^4^ Frontiers Science Center for Synthetic Biology and Key Laboratory of Systems Bioengineering (Ministry of Education), Tianjin University, Tianjin, China

**Keywords:** freezing-tolerant, high sensitivity, stretchability, conductive hydrogels, strain sensor

## Abstract

Currently, stretchable hydrogel has attracted great attention in the field of wearable flexible sensors. However, fabricating flexible hydrogel sensor simultaneously with superstretchability, high mechanical strength, remarkable self-healing ability, excellent anti-freezing and sensing features *via* a facile method remains a huge challenge. Herein, a fully physically linked poly(hydroxyethyl acrylamide)-gelatin-glycerol-lithium chloride (PHEAA-GE-Gl-LiCl) double network organohydrogel is prepared *via* a simple one-pot heating-cooling-photopolymerization method. The prepared PHEAA-GE-Gl-LiCl organohydrogel exhibits favorable stretchability (970%) and remarkable self-healing property. Meanwhile, due to the presence of glycerol and LiCl, the PHEAA-GE-Gl-LiCl organohydrogel possesses outstanding anti-freezing capability, it can maintain excellent stretchability (608%) and conductivity (0.102 S/m) even at −40°C. In addition, the PHEAA-GE-Gl-LiCl organohydrogel-based strain sensor is capable of repeatedly and stably detecting and monitoring both large-scale human motions and subtle physiological signals in a wide temperature range (from −40°C to 25°C). More importantly, the PHEAA-GE-Gl-LiCl organohydrogel-based sensor displays excellent strain sensitivity (GF = 13.16 at 500% strain), fast response time (300 ms), and outstanding repeatability. Based on these super characteristics, it is envisioned that PHEAA-GE-Gl-LiCl organohydrogel holds promising potentials as wearable strain sensor.

## Introduction

Flexible and wearable skin-like strain sensors can convert mechanical deformations into detectable electronic signals (e.g., resistance, capacitance, current, etc.), they have attracted more and more attentions due to the promising potential applications in the field of motion detecting and human-machine interaction (Ge et al., 2018; Xu et al., 2019; Zeng et al., 2019; Zhu et al., 2019; Kim et al., 2020; Kim and Moon, 2020). For example, Qu et al. fabricated a flexible strain sensor based on polydopamine-coated nanocomposites of nitrile rubber and carbon black (Qu et al., 2020). Zhan et al. prepared a high sensitivity of multi-sensing materials based on reduced graphene oxide and natural rubber (Zhan et al., 2021). To meet the requirements of applications, the strain sensors should have desired stretchability, mechanical strength, self-healing ability and strain sensitivity (Xin et al., 2016; Lei et al., 2017; Si et al., 2017; Wang L. et al., 2019; Jing et al., 2019; Kweon et al., 2019). However, the conventional strain sensors prepared by integrating conductive components (e.g., conductive polymers, metal materials, carbon-based materials, etc.) into elastomer (e.g., polyethylene terephthalate, polyurethane, etc.) show poor stretchability (Lee et al., 2014; Kim et al., 2015; Lee et al., 2015; Kim et al., 2018; Zhou et al., 2018). Therefore, it is very important to choose intrinsically highly stretchable conductive materials for fabricating high-performance wearable sensors.

Recently, ionic double network (DN) hydrogels by introducing salt ions (e.g., LiCl, NaCl, KCl, Fe^3+^, Ca^2+^, etc.) have drawn special interest to fabricate flexible and stretchable strain sensors, as they are stretchable and can maintain bulk conductivity during large reversible deformation (Liu and Li, 2017; Cheng et al., 2019; Hou et al., 2019; Yang and Yuan, 2019; Liu et al., 2020; Zhu et al., 2020). For example, Hou et al. fabricated a polyacrylamide (PAAm)-agarose-NaCl hydrogel-based strain sensor with high sensitivity *via* chemically crosslinked PAAm network and physically crosslinked agarose network (Hou et al., 2019). Liu et al. prepared a stretchable strain sensor based on PAAm-carrageenan-KCl DN hydrogel *via* chemically crosslinked PAAm network and physically crosslinked carrageenan network, which could monitor and distinguish various human motions (Liu and Li, 2017). Owing to the existence of Na^+^, K^+^, Ca^2+^ ions in gellan gum (GG), Liu et al. engineered a highly stretchable PAAm-GG hydrogel-based sensor for human motion monitoring *via* chemically crosslinked PAAm network and physically crosslinked GG network (Liu et al., 2020). However, due to the intrinsic irreversibility of the covalent crosslinking, these reported conductive DN hydrogel had unsatisfactory self-healing ability, leading to low durability as the sensor. Therefore, developing a conductive DN hydrogels crosslinked by physical interactions may be an effective way to prepare the flexible strain sensors (Liu et al., 2017; Liu et al., 2018; Chen et al., 2019; Xia et al., 2019; Zhang et al., 2020).

Water-based ionic hydrogels will inevitably freeze at subzero temperatures, which further decrease the stretchability and conductivity properties. Recently, some polyol agents (e.g., glycol, glycerol, sorbitol, etc.) has been introduced into hydrogels to improve the freezing-tolerant property of hydrogel (Liao et al., 2019; Tu et al., 2019; Wu et al., 2019; Pan et al., 2020; Peng et al., 2020). These anti-freezing agents can effectively reduce the aggregation of water molecules and weaken their internal hydrogen bonding, resulting in preventing the formation of ice crystalline and decreasing the freezing points of hydrogels. Based on this point of view, Liao et al. developed an anti-freezing MXene/PAAm-PVA organohydrogel by immersing hydrogel in glycol solution, the prepared organohydrogel exhibited outstanding anti-freezing property at −40°C (Liao et al., 2019). Nevertheless, the immersion procedure is often involved in this strategy, which is laborious and further limiting their large-scale application. Therefore, constructing anti-freezing hydrogels *via* a facile “one-pot” method was more desired. Based on this idea, Peng et al. constructed an anti-freezing PVA-PEDOT/PSS DN organohydrogel by directly using glycol-water binary solvent as dispersion medium (Peng et al., 2020). Pan et al. prepared a poly(vinyl alcohol) hydrogel by dissolving poly(vinyl alcohol) in glycerol-water binary solvent, followed by soaking it in saturated NaCl aqueous solution, the obtained organohydrogel-based sensor could maintain good strain-sensitive performance at −20°C (Pan et al., 2020). However, these hydrogels could not heal once damaged. In consequence, it is highly anticipated to find a facile “one-pot” method to realize an ionic conductive hydrogel simultaneously with integrated superstretchability, high mechanical strength, remarkable self-healing ability, excellent anti-freezing behaviors and sensing features to broaden their application range in spite of the great progress in hydrogel sensors.

Herein, a fully physically crosslinked poly(hydroxyethyl acrylamide)-gelatin-glycerol-lithium chloride (PHEAA-GE-Gl-LiCl) organohydrogel was constructed by a simple one-pot method. It was expected that it had superstretchability, high mechanical strength, remarkable self-healing ability, excellent anti-freezing behaviors and sensing features. The networks of the organohydrogel were formed *via* hydrogen bonds. Incorporation of Li^+^ and Cl^−^ ions imparted PHEAA-GE-Gl-LiCl organohydrogel prominent ionic conductivity. Owing to the presence of glycerol and LiCl, the PHEAA-GE-Gl-LiCl organohydrogel possessed excellent anti-freezing and ultra-high strain sensitivity features, which could be designed as wearable sensors for strain detection in wide temperature ranges (from −40°C to 25°C).

## Materials and Methods

Hydroxyethyl acrylamide (HEAA), gelatin (GE, gel strength ∼240 g bloom), 2-hydroxy-4′-(2-hydroxyethoxy)-2-methylpropiophenone (Irgacure 2959), glycerol (Gl), and lithium chloride (LiCl) were purchased by Shanghai Aladdin Reagent Co. Ltd. Water used in this study was purified by a Millipore water purification system with a resistivity of 18.2 MΩ cm.

### Preparation and Characterization of PHEAA-GE-Gl-LiCl Organohydrogel

The PHEAA-GE-Gl-LiCl organohydrogel was prepared *via* a simple one-pot heating-cooling-photopolymerization method. Briefly, all reactants, including HEAA (36 wt%), GE (4 wt%), initiator Irgacure 2959 (1 mol% of HEAA) and LiCl (1.2 wt%), were added into the mixture solution of glycerol/water (1:3, w/w). Then, the mixture was heated up to 60°C and was injected into a glass mold. After cooling to room temperature, it was exposed to UV light (365 nm, 8 W) for 2 h to form physically cross-linked network of PHEAA. Then, it was stored in a 4°C for 30 min to strengthen gelatin network and the PHEAA-GE-Gl-LiCl organohydrogel was obtained.

The chemical structure of the gels was investigated by Thermo Scientific Nicolet iS10 FT-IR spectrophotometer using an attenuated total reflectance Fourier Transform Infrared (ATR-FTIR) in the 4,000–650 cm^−1^ range (resolution: 4 cm^−1^, number of scans: 32). Before test, the gel samples were prepared into a thin sheet by drying in a freeze dryer.

### Mechanical Properties Tests

The PHEAA-GE-Gl-LiCl organohydrogel samples were cut into dumbbell shape specimens. The mechanical properties were measured using a tensile tester (Instron 3369, United States) at 100 mm/min. For the tensile tests, the nominal stress (*σ*) was calculated as the loading force divided by the original average cross-sectional area of the sample. The nominal strain (*ε*) was defined as the resultant length divided by the starting length of the sample. The elastic modulus (E) was obtained from the slope of the stress-strain curve in the elastic region (0–15% strain). The toughness was calculated by integrating the area below the stress-strain curve. The dissipated energy was calculated by the enclosed area of the integral curve between the loading-unloading curves.

### Self-Healing Behaviors Test

The rectangular-shaped PHEAA-GE-Gl-LiCl organohydrogels with and without staining were cut into two pieces, respectively. Then, the cut surfaces were brought together manually and sealed in a polyethylene bag. Subsequently, the sealed PHEAA-GE-Gl-LiCl organohydrogels were stored at 25°C or 60°C. After different time intervals, the self-healed gel samples were investigated by tensile test. The self-healing efficiency of the gel samples was evaluated by stress-strain curves of the original and the self-healed PHEAA-GE-Gl-LiCl organohydrogel. The self-healing efficiency (HE) was defined according to Eq. 1,

$$\mathrm{HE}=\sigma_{t}\sigma_{0}\times 100\%\;,$$ (1)

where σ_0_ is the tensile stress of the original PHEAA-GE-Gl-LiCl organohydrogel, while σ_t_ is the tensile stress of the self-healed PHEAA-GE-Gl-LiCl organohydrogel under different temperatures or different healing time.

### Determination of Anti-Freezing Property

The anti-freezing ability of the gel samples was characterized *via* differential scanning calorimetry (DSC) (DSC 25, TA Instruments) with a refrigerated cooling system (TA Instruments, RCS90), the cooling cycle was executed from 20°C to −70°C at a rate of 2°C min^−1^. Rheological test was performed on a rotational rheometer (DHR-2, TA Instruments) under a fixed strain of 0.1% and fixed frequency of 1 Hz, this temperature-sweep was cooled from 25°C to −40°C at a rate of 2°C min^−1^. The storage modulus (G′) and loss modulus (G″) were recorded. Besides, the stretching behavior of different gels at −40°C was tested using a tensile tester (Instron 3369, United States) with a separate frozen chamber.

### Electrical Measurement

The conductivity of the prepared gel was evaluated by the linear sweep voltammetry method using an electrochemical workstation (Vertex C, IVIUM Tech, Netherlands). The conductivity was calculated according to Eq. 2,

$$\sigma =\frac{1}{\rho }=\frac{l}{R\times A},$$ (2)

where *l* and *A* represent the length and cross-sectional area of the gel, respectively. *R* means the resistance, which was measured by electrochemical workstation. Meanwhile, the conductive behavior of the gel sample at different strain was investigated to obtain the relative resistance changes (*ΔR/R*_*0*_), where *R* and *R*_*0*_ are the resistances of the original and stretched gel sample, respectively. The gauge factor (*GF*) of the gel is defined according to Eq. 3,

$$\mathrm{GF}=\frac{\Delta R/R_{0}}{\Delta l}=\frac{\left(R-R_{0}\right)/R_{0}}{\Delta l}.$$ (3)

### Fabrication and Tests of Strain Sensor

A strain sensor was assembled by using PHEAA-GE-Gl-LiCl organohydrogel. At first, the PHEAA-GE-Gl-LiCl organohydrogel was cut into a stripshaped specimen. Then, two copper wires were fixed at the two ends of the PHEAA-GE-Gl-LiCl organohydrogel specimen to assemble into a strain sensor. VHB tape was used to encapsulate the strain sensor to avoid the water evaporation. For detecting human motion, the organohydrogel based sensor was attached directly on skin surface of the volunteer *via* VHB tape and the sensing performance was evaluated using an electrochemical workstation. For the motion detection of PHEAA-GE-Gl-LiCl organohydrogel-based strain sensor at −40°C, PHEAA-GE-Gl-LiCl organohydrogel-based strain sensor was attached to the wooden human model and prosthetic hand and held in a −40°C environment. The sensing performance for imitated human motions was obtained by manually bending the joint components of the wooden mode.

## Results and Discussion

### Formation of PHEAA-GE-Gl-LiCl Organohydrogel

**FIGURE 1:**
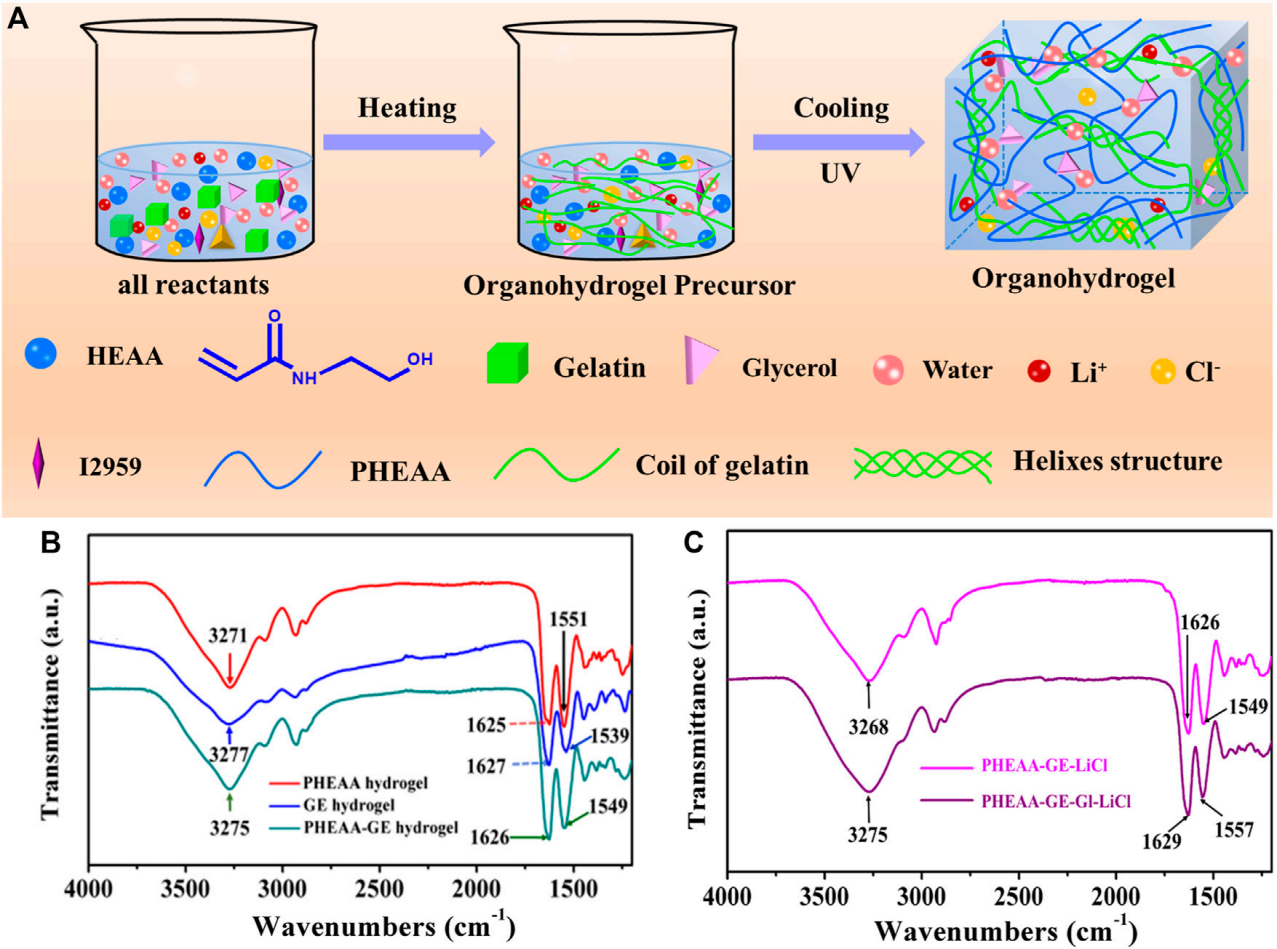
**(A)** Schematic of a one-pot synthesis of a fully physically cross-linked PHEAA-GE-Gl-LiCl organohydrogel. **(B)** ATR-FTIR spectroscopy of PHEAA SN hydrogel, GE SN hydrogel, and PHEAA-GE DN hydrogel. **(C)** ATR-FTIR spectroscopy of PHEAA-GE-LiCl hydrogel and PHEAA-GE-Gl-LiCl organohydrogel.

Figure 1A displayed the fabrication procedure of the PHEAA-GE-Gl-LiCl organohydrogel *via* a simple one-pot heating-cooling-photopolymerization method. At first, all reactants including UV initiator Irgacure 2959, HEAA, GE, glycerol and LiCl were dispersed in deionized water. Subsequently, the mixture solution was heated to 60°C to dissolve GE, and then was cooled to room temperature. Then, it was exposed to UV light to initiate the polymerization of HEAA monomer and form the physical crosslinked PHEAA network *via* the hydrogen bonding among hydroxyl groups of PHEAA chains, which interpenetrated with the GE network, and finally formed PHEAA-GE-Gl-LiCl organohydrogel with a milky white color. During this preparation process, no chemical cross-linked structure was introduced. The prepared PHEAA-GE-Gl-LiCl organohydrogel was formed *via* the reversible noncovalent bonds.

ATR-FTIR spectra were used to investigate the interactions in PHEAA-GE-Gl-LiCl organohydrogel. As shown in Figure 1B, PHEAA hydrogel exhibited the absorption band at 3,271 cm^−1^, which was attributed to the stretching vibration of O-H and N-H. Meanwhile, the two characteristic peaks at 1,625 cm^−1^ and 1,551 cm^−1^ were assigned to the stretching vibration of C=O and bending vibration of N-H from amide, respectively. For GE hydrogel, the broadband at 3,277 cm^−1^ was assigned to the N-H stretching vibrations, and the characteristic peaks at 1,627 cm^−1^ and 1,539 cm^−1^ were assigned to the stretching vibration of C=O and bending vibration of N-H from amide, respectively. Compared with PHEAA hydrogel and GE hydrogel, few new absorption bands appear in the PHEAA-GE hydrogel, indicating there were no chemical reactions between PHEAA and GE networks. Meanwhile, the stretching vibrations of O-H and N-H, stretching vibration of C=O and bending vibration of N-H from amide shifted to 3,275, 1,626, and 1,549 cm^−1^, respectively, suggesting the formation of hydrogen bonds between the PHEAA networks and GE networks. To illustrate the interactions between glycerol and PHEAA-GE-LiCl network, ATR-FTIR of PHEAA-GE-LiCl hydrogel and PHEAA-GE-Gl-LiCl organohydrogel was also analyzed. As presented in Figure 1C, after addition glycerol, the stretching vibration of O-H and N-H (3,268 cm^−1^), stretching vibration of C=O (1,626 cm^−1^), and bending vibration of N-H (1,549 cm^−1^) in PHEAA-GE-LiCl hydrogel shifted to 3,275, 1,629, and 1,557 cm^−1^ in the PHEAA-GE-Gl-LiCl organohydrogel, respectively, indicating the formation of hydrogen bonds between glycerol molecules and the gel networks owing to the -OH and -NH- groups among PHEAA, GE and glycerol.

### Mechanical Properties of PHEAA-GE-Gl-LiCl Organohydrogel

**FIGURE 2:**
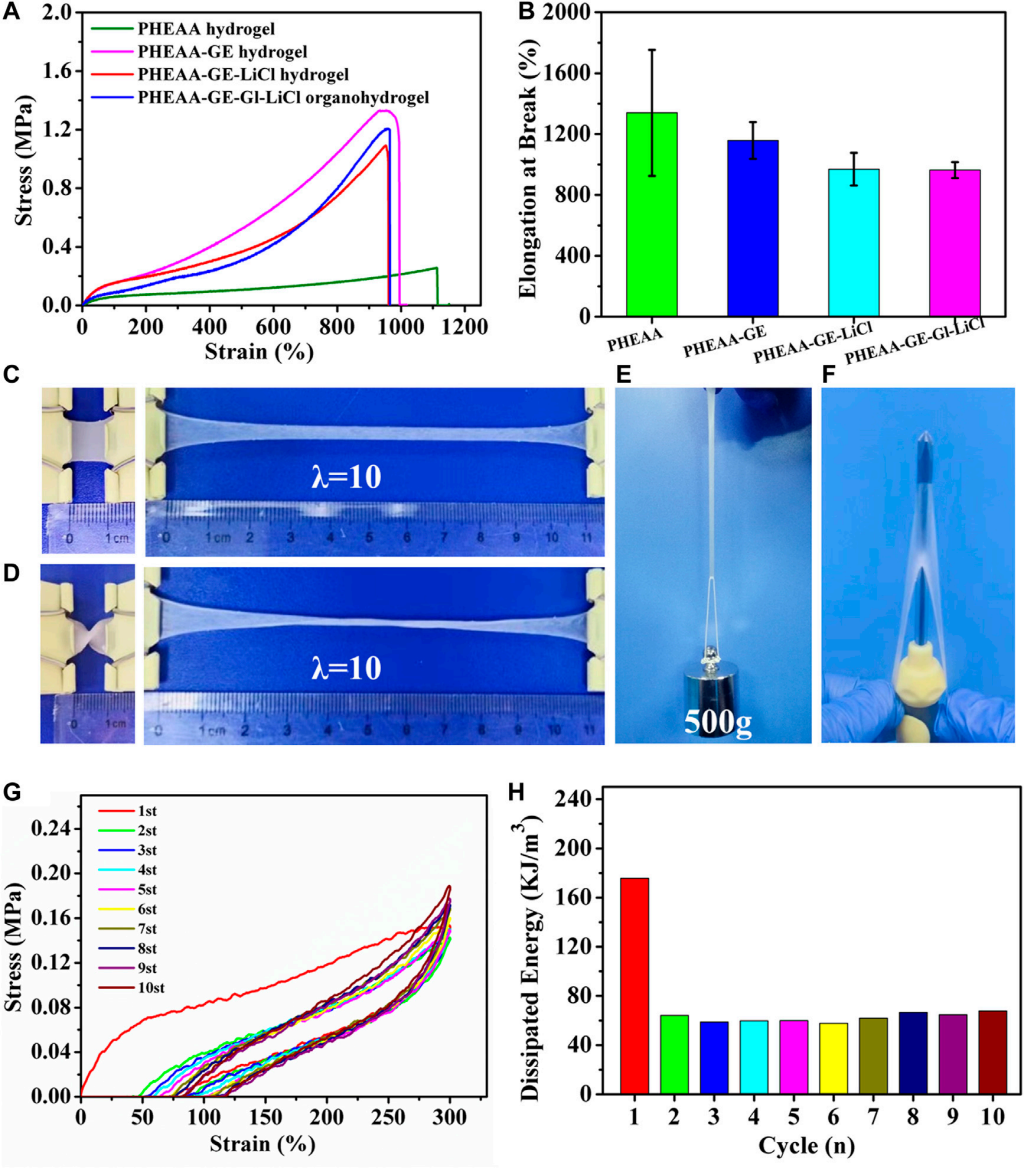
The mechanical property of PHEAA-GE-Gl-LiCl DN organohydrogel. **(A)** The typical tensile stress-strain curves of PHEAA hydrogel (C_PHEAA_ = 40 wt%), PHEAA-GE hydrogel (C_PHEAA_ = 36 wt%, C_GE_ = 4 wt%), PHEAA-GE-LiCl hydrogel (C_PHEAA_ = 36 wt%, C_GE_ = 4 wt%, C_LiCl_ = 1.2 wt%), PHEAA-GE-Gl-LiCl DN organohydrogel [C_PHEAA_ = 36 wt%, C_GE_ = 4 wt%, C_LiCl_ = 1.2 wt%, and Gl:water (w/w) = 1:3], and **(B)** their corresponding elongations at break. Photos of PHEAA-GE-Gl-LiCl DN organohydrogel at different states of **(C)** stretching, **(D)** curly stretching, **(E)** sustaining a weight of 500 g and **(F)** puncture resistance. **(G)** Ten continuous cyclic stretching and releasing process of the PHEAA-GE-Gl-LiCl organohydrogel under 300% tensile strain and **(H)** the corresponding hysteresis energy in every cycle.

The mechanical property of PHEAA-GE-Gl-LiCl organohydrogel was primarily dependent on the network composition and structure. Figures 2A,B showed the typical tensile stress-strain curves of different hydrogels/organohydrogels and the corresponding elongation at break. Compared with the PHEAA hydrogel (0.23 MPa), the stress of PHEAA-GE hydrogel remarkably enhanced to 1.44 MPa. After introducing LiCl and glycerol, the stress appeared to decrease slightly. The elasticity modulus of PHEAA hydrogel, PHEAA-GE hydrogel, PHEAA-GE-LiCl hydrogel, and PHEAA-GE-Gl-LiCl organohydrogel was 0.10, 0.29, 0.30, and 0.22 MPa, respectively. Besides, all these hydrogels exhibited high fracture strain in the range of 1,158%–964% (Figure 2B). Figures 2C,D visually illustrated PHEAA-GE-Gl-LiCl organohydrogel was flexible and tough, it could be easily stretched to approximately 10 times of its original length without rupture even at twisted state. In addition, the PHEAA-GE-Gl-LiCl organohydrogel was strong enough to sustain a weight of 500 g (625 times of its own weight) without breaking (Figure 2E) and was tough enough to resist to the puncture (Figure 2F). Supplementary Figure S1 indicated PHEAA-GE-Gl-LiCl organohydrogel displayed a very smooth and flat structure, which was possibly because noncovalent interactions within the PHEAA-GE-Gl-LiCl organohydrogel promoted the connected network.

To better illustrate the mechanical properties of the prepared organohydrogel, the effects of the solid content of PHEAA, the weight ratios of PHEAA to GE, the amount of LiCl and glycerol on the mechanical properties of hydrogels were investigated by uniaxial tensile tests. As shown in Supplementary Figure S2, a minimum concentration of HEAA monomers (20 wt%) was required to form PHEAA SN hydrogels. With the enhancement of PHEAA concentration from 25 to 50 wt%, the tensile strength of PHEAA SN hydrogels increased from 0.14 to 0.32 MPa and the elongation at break of all the PHEAA SN hydrogels was higher than 800%, indicating PHEAA network was soft and tough (Supplementary Figure S3). The mechanical property of PHEAA-GE hydrogel was remarkably enhanced compared with PHEAA SN hydrogel (Figure 2A). Meanwhile, as the weight ratios of PHEAA/GE decreased from 19:1 to 3:1, the tensile strength of PHEAA-GE hydrogel continuously increased from 0.69 to 2.31 MPa, while both of elongation at break and toughness increased first and then decreased. At the weight ratio of 9:1, the maximum fracture strain (1,158%) and toughness (8.17 MJ/m^3^) were achieved (Supplementary Figure S4). Owing to the rigid and brittle nature of the GE network, the higher concentration of GE made PHEAA-GE hydrogel stronger but more brittle. Besides, the introduction of LiCl or glycerol can also impact the mechanical property (Supplementary Figure S5, Supplementary Figure S6). With the enhancement of LiCl concentration from 0.2 to 1.6 wt%, the tensile strength decreased from 1.26 to 0.74 MPa, and the elongation at break decreased from 1,238 to 806%, which was likely due to the destruction of hydrogen bonds between PHEAA networks and/or GE networks. Furthermore, there were large amounts of -C=O and -NH groups in the hydrogel matrix, so the interaction between PHEAA networks and/or GE networks might be influenced when glycerol replaced part of water. As presented in Supplementary Figure S6, with an increase in the proportion of glycerol/water from 1:4 to 1:2, the tensile stress decreased from 1.37 to 0.76 MPa and the fracture strain increased from 974 to 1,274%, which indicated the introduction of glycerol weakened the interactions between PHEAA networks and/or GE networks and meanwhile enhanced the gel flexibility.

PHEAA-GE-Gl-LiCl organohydrogel was formed *via* the fully reversible non-covalent bonds, so it was expected that the PHEAA-GE-Gl-LiCl organohydrogel had energy dissipation and self-recovery ability. As displayed in Figure 2G, a successive cyclic tensile test at a maximum strain of 300% without resting time between each cycle was conducted. Obviously, the PHEAA-GE-Gl-LiCl organohydrogel exhibited a large hysteresis loop in the first cycle, indicating plenty of energy could be dissipated by rapid dissociation of physical interactions. From the second to 10th cycles, the corresponding dissipated energies remained almost unchanged at around 62 KJ/m^3^ (Figure 2H), indicating the good fatigue resistance of PHEAA-GE-Gl-LiCl organohydrogel due to the rapid self-recoverability (Qin et al., 2020).

### Healing Performances of PHEAA-GE-Gl-LiCl Organohydrogel

**FIGURE 3:**
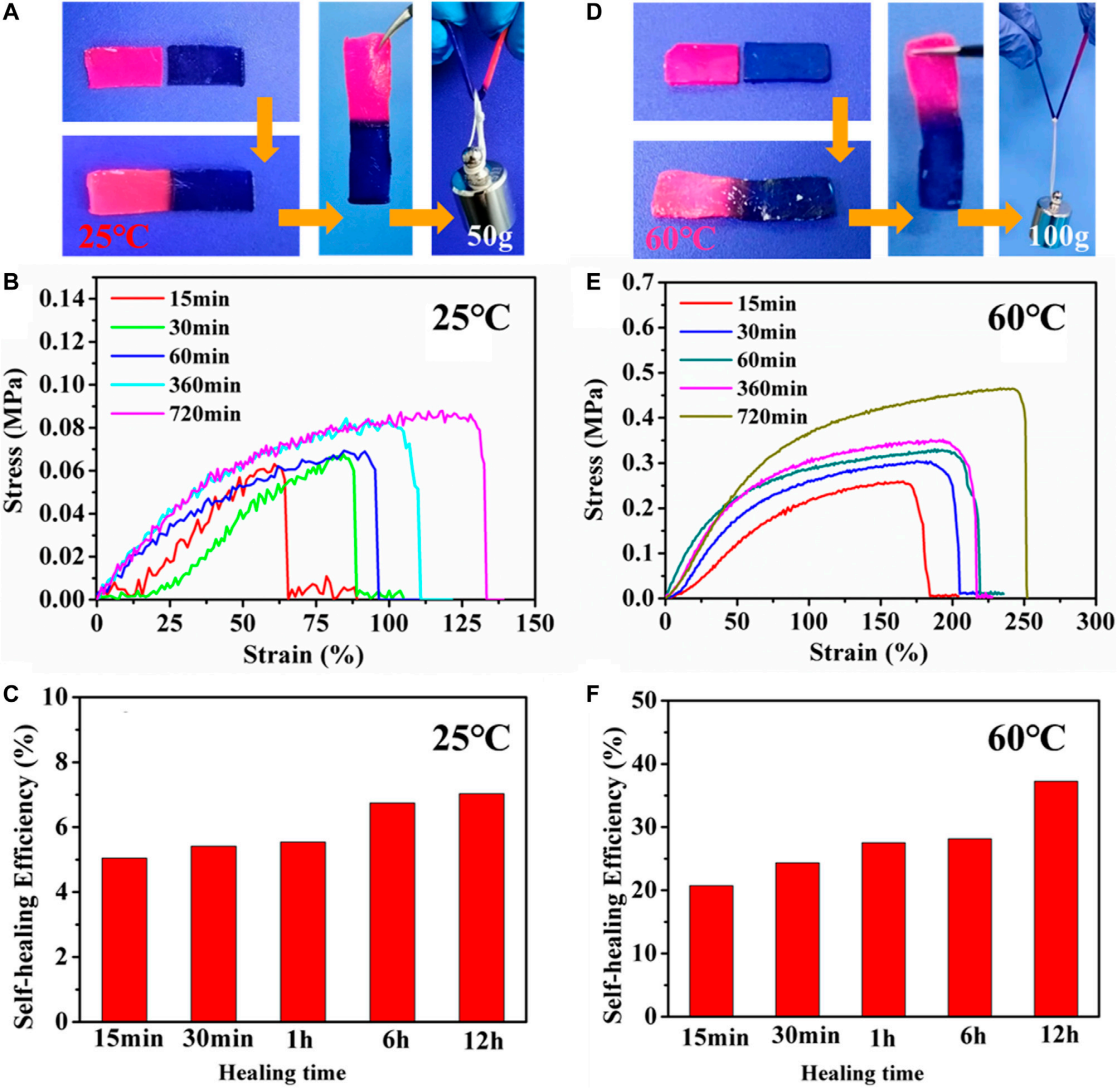
The self-healing property of PHEAA-GE-Gl-LiCl organohydrogel. The self-healed PHEAA-GE-Gl-LiCl organohydrogel **(A)** at 25°C for 12 h and **(D)** at 60°C for 12 h could withstand a weight of 50 and 100 g, respectively. Stress-strain curve and self-healing efficiency of the self-healed PHEAA-GE-Gl-LiCl organohydrogel at different healing time **(B,C)** at 25°C and **(E,F)** at 60°C.

Due to the reversible non-covalent interactions in PHEAA-GE-Gl-LiCl organohydrogel, good self-healing property of PHEAA-GE-Gl-LiCl organohydrogel was expected. As shown in Figure 3A, when two pieces of PHEAA-GE-Gl-LiCl organohydrogel (red and blue) were in contact with each other, the obvious interface could be observed. After storing at room temperature (25°C) for 12 h, the two hydrogels fused together, and the interface became indistinct. Moreover, the healed PHEAA-GE-Gl-LiCl organohydrogel could sustain a load of 50 g (55 times of its own weight), implying PHEAA-GE-Gl-LiCl organohydrogel possessed good self-healing property at room temperature. To quantify the self-healing property, the tensile stress-strain curves of the healed PHEAA-GE-Gl-LiCl organohydrogel were investigated. As shown in Figure 3B, the stress and elongation at break increased with the increase of healing time. The tensile strength and elongation at break of healed PHEAA-GE-Gl-LiCl organohydrogel for 12 h was 0.09 MPa and 139%, respectively. The healing efficiency was 7.02% at 12 h (Figure 3C). At 25°C, the self-healing property was attributed to the facile breaking and reforming of hydrogen bonds originated from the self-association of the PHEAA network. This performance was unlike with PAAm-based hydrogels crosslinked *via* covalent bond, whose self-healing property could not be achieved at 25°C because the fractured chemically cross-linked PAAm network was unable to be reconstructed. Usually, to realize the self-healing ability of PAAm-based hydrogels, additional heat treatment must be needed to promote the movement of polymeric chain in network. However, even so, the healing efficiency was not satisfying.

The triple-helix bundles of GE can be transformed into coils when the temperature is high than its conformational transition temperature, then the triple-helix bundles can be re-associated when temperature decreases (Chen et al., 2017; He et al., 2018; Qin et al., 2020). Therefore, the self-healing ability of PHEAA-GE-Gl-LiCl organohydrogel could be enhanced at 60°C. As displayed in Figure 3D, compared with the healed PHEAA-GE-Gl-LiCl organohydrogel at 25°C for 12 h, the healed PHEAA-GE-Gl-LiCl organohydrogel at 60°C for 12 h could sustain a weight of 100 g. Figures 3E,F showed the stress, fracture strain, and the healing efficiency of the healed PHEAA-GE-Gl-LiCl at 60°C was much higher than those at 25°C at the same healing time. Upon the heating treatment at 60°C for 12 h, the tensile stress, fracture strain, and the healing efficiency was 0.47 MPa, 263%, and 37.23%, respectively, which was much higher than those of the healed PHEAA-GE-Gl-LiCl organohydrogel at 25°C. The schematic diagram of the self-healing mechanism was shown in Supplementary Figure S7. On the whole, due to the collaborative results of two physical crosslinked networks, the healing behaviors of PHEAA-GE-Gl-LiCl organohydrogel is much higher than all the reported self-healed PAAm-based or PHEAA-based hydrogel sensor (Supplementary Table S1).

### Anti-Freezing Property of PHEAA-GE-Gl-LiCl Organohydrogels

Owing to large amounts of water, ionically conductive hydrogels will be frozen and lose stretchable property. Meanwhile, the conductivity also obviously decreases owing to the low movement capacity of the free ions at subzero temperatures (Chen et al., 2018; Rong et al., 2018). Therefore, the water-based hydrogel cannot be used under subzero temperature. In this work, the glycerol and LiCl were introduced into the hydrogel network to improve the freezing tolerances.

**FIGURE 4:**
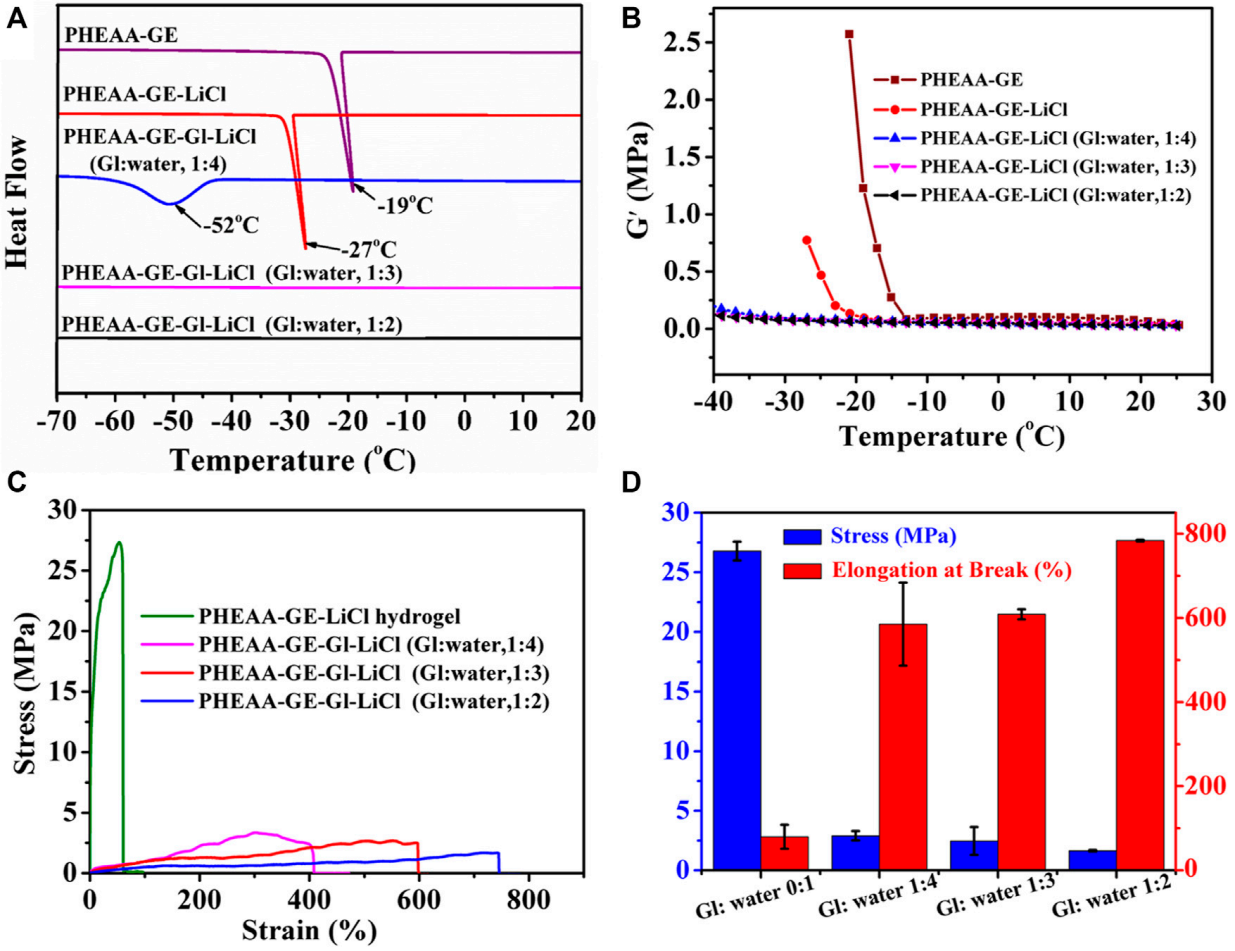
Anti-freezing properties of PHEAA-GE-Gl-LiCl organohydrogels. **(A)** DSC curves of hydrogel and organohydrogel with different Gl/water ratios (0:1, 1:4, 1:3, and 1:2). **(B)** Storage modulus (G′) of hydrogel and organohydrogel with different Gl/water ratios (0:1, 1:4, 1:3, and 1:2) at the temperature range of 25°C to −40°C. The **(C)** stress-strain curves, **(D)** stress and elongation at break of PHEAA-GE-LiCl hydrogel and PHEAA-GE-Gl-LiCl organohydrogel with different weight ratios of Gl/water at −40°C.

DSC experiment was performed to investigate the ice formation in the organohydrogel. As shown in Figure 4A, a sharp exothermic peak at −19°C appeared in PHEAA-GE hydrogel, indicating large amounts of water was converted to ice at this condition. After addition of LiCl, the exothermic peak reduced to −27°C owing to the colligative property of ionic compounds (Gao et al., 2019), indicating that the introduction of LiCl decreased the freezing point of water. Further, the freezing point was dramatically reduced to −52°C when 20% glycerol was introduced (glycerol/water, 1:4). Surprisingly, the exothermic peak entirely disappeared when the glycerol/water ratio increased to 1:3 and 1:2. These results demonstrated the introduction of glycerol could effectively decrease the freezing point or even completely depress ice generation by the comprehensive effect combined colligative property of LiCl and forming hydrogen bonds between the hydroxyls in the glycerol and water molecules.

The rheological test at temperature ranges from 25°C to −40°C showed a similar phenomenon (Figure 4B). The G′ value of the PHEAA-GE abruptly increased when the temperature fell below −13°C. At this temperature, the ice crystals started to grow and PHEAA-GE-LiCl hydrogel began to transform into an ice-like solid. The introduction of LiCl decreased this behavior, and the G′ value of PHEAA-GE-LiCl hydrogel abruptly increased when temperature fell below −20°C. In contrast, the G′ value of PHEAA-GE-Gl-LiCl organohydrogels (1:4, 1:3, and 1:2) showed persistent stability within the test temperature range (from 25°C to −40°C), indicating that the introduction of glycerol was beneficial to the maintenance of the flexible feature of PHEAA-GE-Gl-LiCl organohydrogels at subzero temperature. To further verify this result, the stretching behavior of the different gels was also investigated at −40°C. As shown in Figures 4C,D, compared with the stress (26.78 MPa) and elongation at break (80%) of PHEAA-GE-LiCl hydrogel, all the PHEAA-GE-Gl-LiCl organohydrogel possessed lower stress (1.65–2.91 MPa) and higher fracture strain (585–784%) (Supplementary Video S1). This behavior was caused by the existing glycerol in the organohydrogel, which hindered the crystallization of water and enhanced the anti-freezing property. Meanwhile, as the glycerol content increased, the stress decreased and elongation at break increased. The fracture strain was much higher than most of the previously reported anti-freezing hydrogel-based gel (Supplementary Table S2). On the whole, on account of the presence of both glycerol and LiCl, PHEAA-GE-Gl-LiCl organohydrogel possessed outstanding anti-freezing capability, which could effectively expand its application in low-temperature environments.

### Conductivity of PHEAA-GE-Gl-LiCl Organohydrogels

**FIGURE 5:**
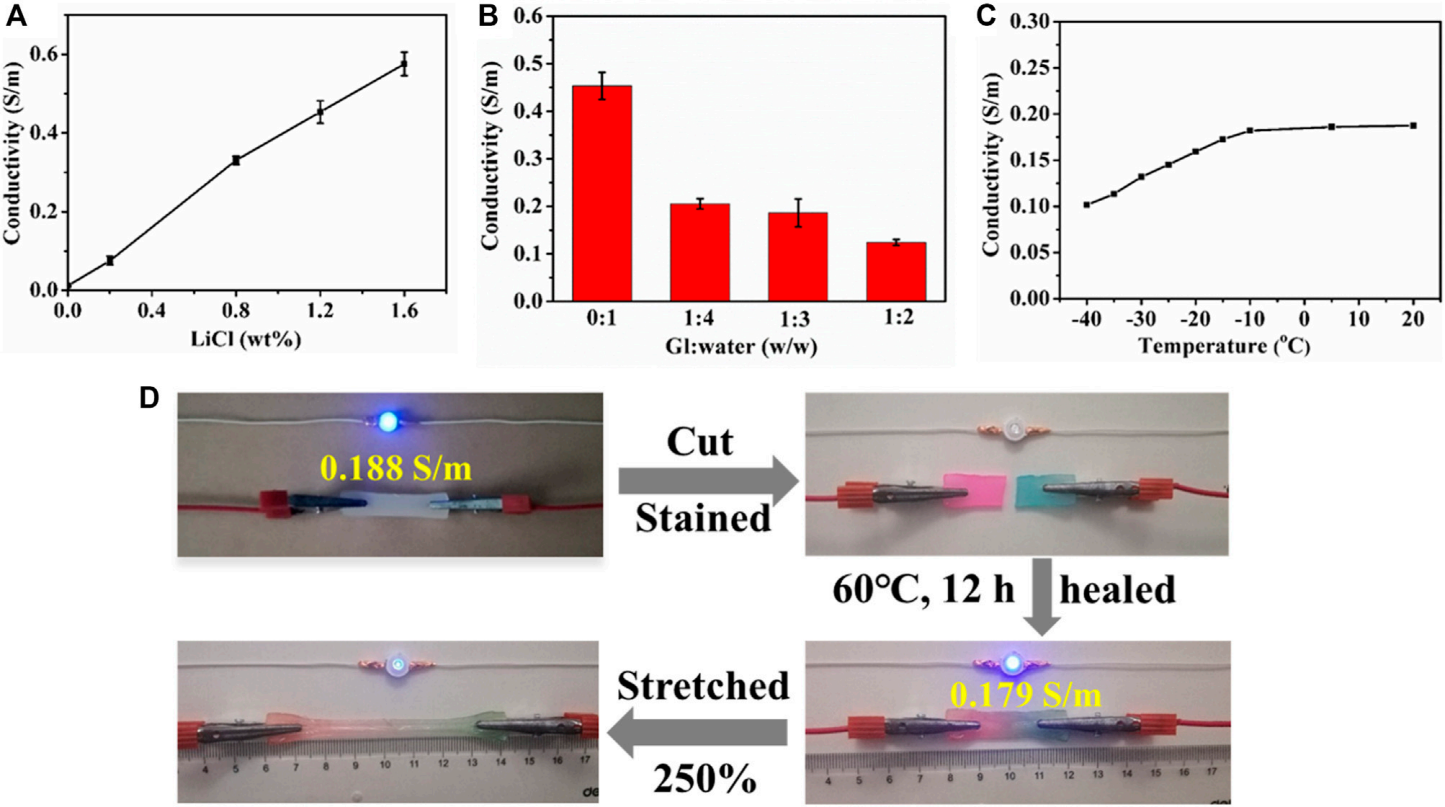
The conductivity of PHEAA-GE-Gl-LiCl organohydrogels. Effect of **(A)** LiCl concentration, **(B)** the weight ratios of Gl/water, **(C)** temperature, and **(D)** circuit comprises an LED indicator connected by original, cut, self-healed, stretched self-healed PHEAA-GE-Gl-LiCl organohydrogel sheets.

To improve the conductivity, LiCl was introduced into PHEAA-GE-LiCl hydrogel. As shown in Figure 5A, with the enhancement of LiCl concentration from 0 to 1.6 wt%, the conductivity rapidly increased from 0.012 to 0.576 S/m. The glycerol/water ratio can also modulate the conductivity of PHEAA-GE-Gl-LiCl organohydrogel, the conductivity decreased from 0.454 to 0.124 S/m when the glycerol/water ratios increased from 0:1 to 1:2 (Figure 5B). This phenomenon was because the migration velocity of free ions (Li^+^ and Cl^−^) in the PHEAA-GE-Gl-LiCl organohydrogel reduced owing to the high viscosity characteristics of glycerol (Wang Q. et al., 2019; Yu et al., 2021). In addition, the temperature also impacted the conductivity of PHEAA-GE-Gl-LiCl organohydrogel. As displayed in Figure 5C, when the temperature was above −10°C, the conductivity of PHEAA-GE-Gl-LiCl organohydrogel changed slightly. Once the temperature dropped to −10°C, the conductivity decreased with the decrease of temperature, which was because the free ions were more difficult to move in the network at low temperature (Yang et al., 2021). Even so, PHEAA-GE-Gl-LiCl organohydrogel also possessed good conductivity (0.102 S/m) at −40°C.

The conductive activity of the healed PHEAA-GE-Gl-LiCl organohydrogel was investigated *via* connecting in the circuit with a blue LED bulb. As shown in Figure 5D, the LED bulb was successfully lit when a voltage of 4.5 V was applied. When PHEAA-GE-Gl-LiCl organohydrogel was cut, the conductive circuit was disconnected, and thus the LED bulb was extinguished. Subsequently, the two fractured surfaces were stained and contacted to heal at 60°C for 12 h, the LED bulb was lit again due to the self-healing capacity of PHEAA-GE-Gl-LiCl organohydrogel. The conductivity of the healed PHEAA-GE-Gl-LiCl organohydrogel recovered to 95.21% of its original state. When the healed PHEAA-GE-Gl-LiCl organohydrogel was pulled to 2.5 times of its original length, the brightness of the LED bulb darkened obviously, signifying that the self-healed PHEAA-GE-Gl-LiCl organohydrogel still possessed outstanding strain-sensitive behaviors.

### Strain Sensor Based on PHEAA-GE-Gl-LiCl Organohydrogel for Detection of Human Motions

**FIGURE 6:**
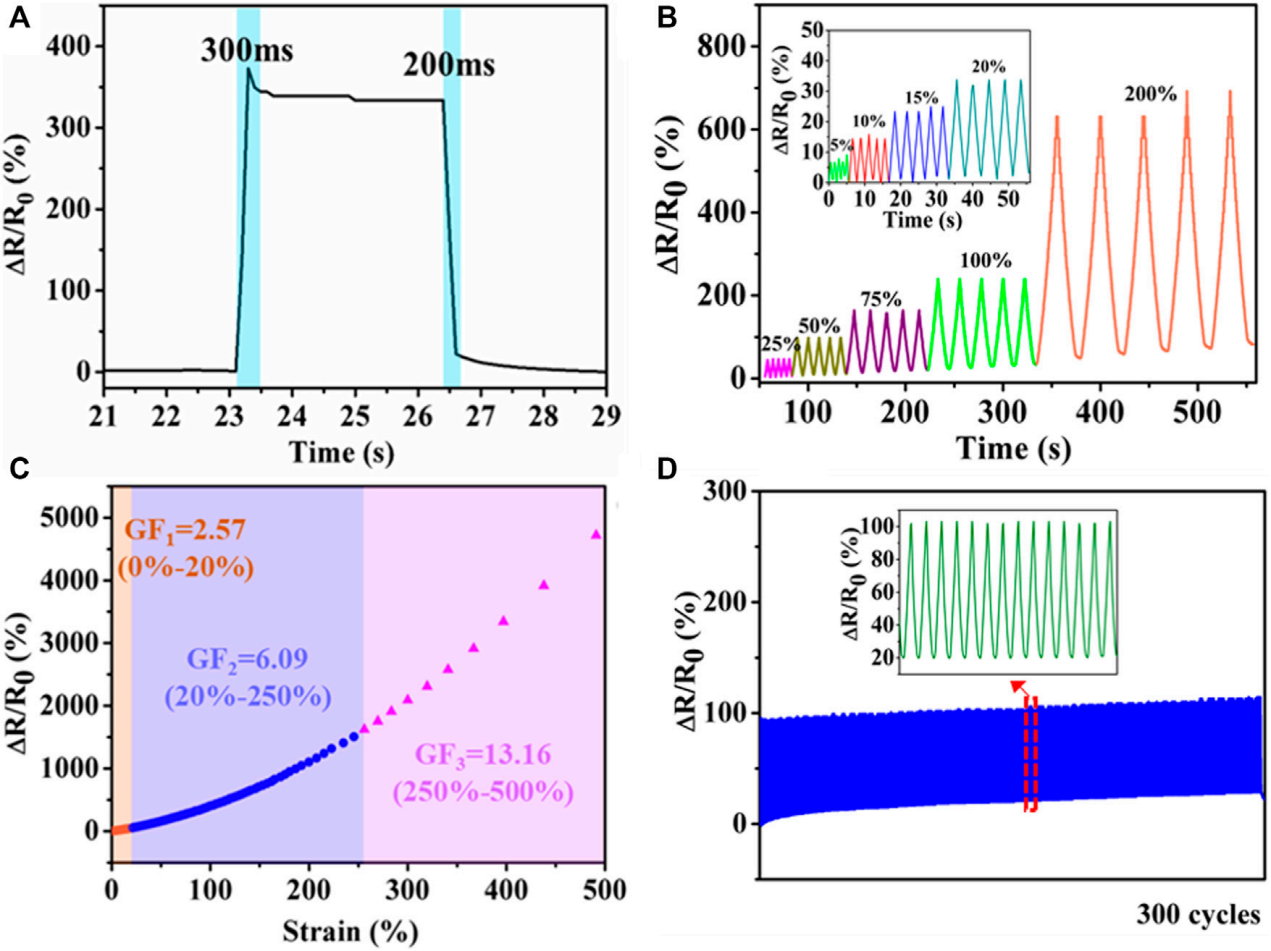
Strain sensing behavior of PHEAA-GE-Gl-LiCl organohydrogel. **(A)** Response time, **(B)** the relative resistance under different small reciprocating strains (5, 10, 15, and 20%) and large reciprocating strains (25, 50, 75, 100, and 200%), **(C)** relative resistance changes and corresponding gauge factors variation with successive tensile strain from 0 to 500%, **(D)** the relative resistance change under cyclic loading of 50% for 300 cycles.

Responsivity is one of the most important parameters for sensors. Here, the response and recovery time was defined as the time interval during which relative resistance variation changed from one stable value to another in response to an instantaneous stretching and releasing deformation. As displayed in Figure 6A, the response and recovery time of PHEAA-GE-Gl-LiCl organohydrogel was 300 and 200 ms, respectively, demonstrating that PHEAA-GE-Gl-LiCl organohydrogel-based strain sensor possessed a rapid response and recovery ability. Figure 6B presented PHEAA-GE-Gl-LiCl organohydrogel-based sensor could sensitively detect both small strain (5–20%) and relatively large strain (25–200%) with good stability and repeatability. And the corresponding relative resistance change (*ΔR/R*_*0*_) monotonously increased as the strain increased from 0 to 500% (Figure 6C). The obvious increase in resistance during stretching was due to the narrowing of cross-section, which reduced the transportation efficiency of Li^+^ and Cl^−^ ions. The resistance change of PHEAA-GE-Gl-LiCl organohydrogel was also visually observed in Supplementary Video S2, an LED bulb in the circuit displayed alternate luminance variation during stretching and releasing PHEAA-GE-Gl-LiCl organohydrogel under 300% tensile strain. The GF values of PHEAA-GE-Gl-LiCl organohydrogel were 2.57, 6.09, and 13.16 in the 0–20%, 20–250%, and 250–500% strain ranges, respectively. When the strain was 500%, the *ΔR/R*_*0*_ was 13.16%, indicating a high strain sensitivity and broad working range. Notably, the sensitivity of PHEAA-GE-Gl-LiCl organohydrogel-based sensor was much higher than that of previously reported stretchable strain sensors (Supplementary Table S3). Figure 6D displayed PHEAA-GE-Gl-LiCl organohydrogel-based sensor was able to maintain repeatable output signals during 300 consecutive cycles at a strain of 50%.

**FIGURE 7:**
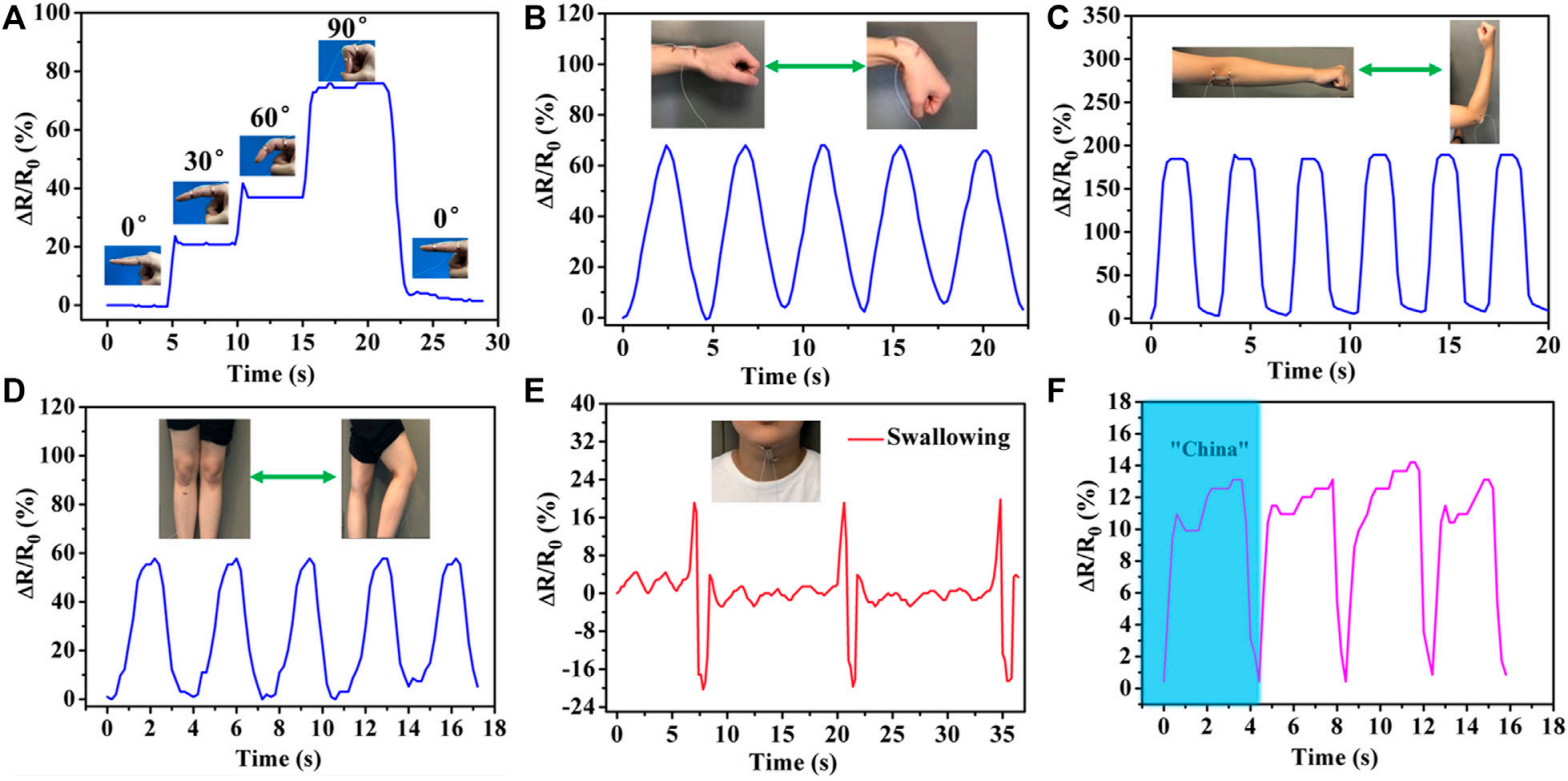
Real-time monitoring of human physical activities based on wearable PHEAA-GE-Gl-LiCl organohydrogel sensor. Relative resistance changes (*ΔR/R*_*0*_) of different human joint motions: **(A)** finger bending at different angles, **(B)** wrist joint bending, **(C)** elbow joint bending, **(D)** knee joint bending. Detection of subtle motions of **(E)** swallowing and **(F)** pronouncing word “China.”

Owing to their favorable stretchability, excellent self-healing ability and high strain sensitivity, the PHEAA-GE-Gl-LiCl organohydrogel showed tremendous potential applications in wearable devices. Here, PHEAA-GE-Gl-LiCl organohydrogel was assembled to be a resistance-type strain sensor to detect full-range human activities in real time. First, the strain sensor was attached on human joints (finger, wrist, elbow, and knee joints) to detect the joint motions. As shown in Figure 7A, when the finger was bent from the straightened state to 30, 60, and 90°, the *ΔR/R*_*0*_ gradually increased to 20.71, 36.91, and 74.36%, respectively. When the finger was held at the same bending angle, the resistance values were consistent. Besides, the resistance value immediately returned to its original level when the finger was straightened. The PHEAA-GE-Gl-LiCl organohydrogel-based strain sensor could also be used to precisely monitor and distinguish the extending/flexing of wrist, elbow, and knee (Figures 7B,C,D). The extending/flexing of elbow displayed the largest relative resistance change due to the largest deformation. Meanwhile, the response signals were repeatable during the cyclic extending/flexing process. Apart from the capacity of monitoring large-scale human motions, the PHEAA-GE-Gl-LiCl organohydrogel-based strain sensor could also perceive subtle deformation. As shown in Figure 7E, the PHEAA-GE-Gl-LiCl organohydrogel-based strain sensor can be fixed on the throat of a volunteer to monitor the swallowing motion. The resistance signals were obvious and stable when the volunteer performed periodic swallowing motions. Furthermore, the PHEAA-GE-Gl-LiCl organohydrogel-based strain sensor could also be used to detect and distinguish pronouncing. The characteristic peaks were similar when the tester pronounced the same word repeatedly, while the PHEAA-GE-Gl-LiCl organohydrogel-based strain sensor showed distinguishable characteristic peaks when saying different words, such as “China” and “Hydrogel” (Figure 7F and Supplementary Figure S8). These results demonstrated that the PHEAA-GE-Gl-LiCl organohydrogel-based strain sensor exhibited great potential as a high-performance wearable device to detect full-range human activities.

### Flexible Sensing Behavior at Subzero Temperature

**FIGURE 8:**
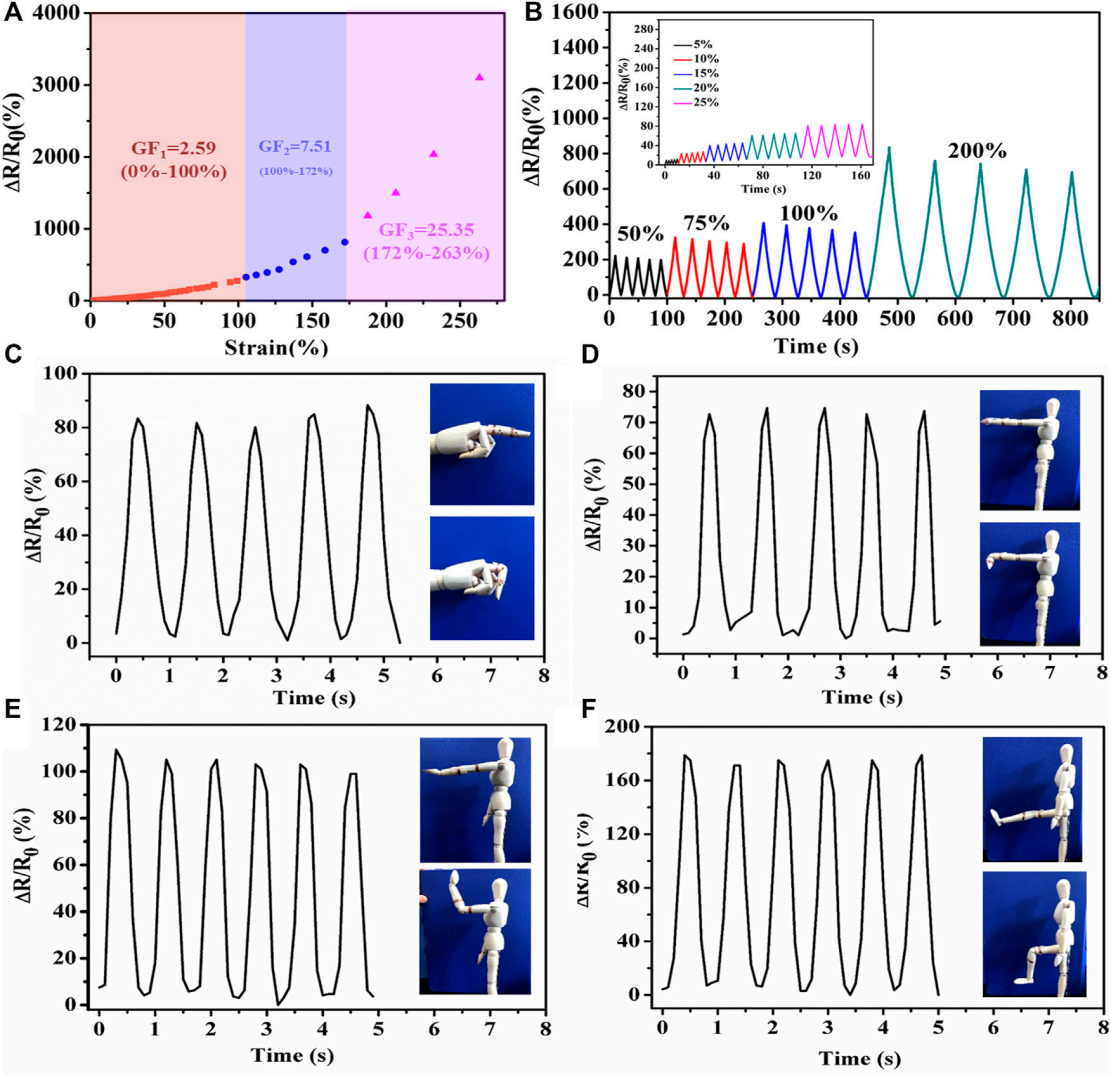
Sensing performance of PHEAA-GE-Gl-LiCl organohydrogel-based strain sensor at −40°C. **(A)** Relative resistance changes and corresponding gauge factors variation with successive tensile strain from 0 to 280%, **(B)** the relative resistance under different small reciprocating strains and large reciprocating strains. Relative resistance changes of imitative human joints motions: **(C)** finger bending, **(D)** wrist joint bending, **(E)** elbow joint bending, and **(F)** knee joint bending at −40°C.

The PHEAA-GE-Gl-LiCl organohydrogel can be used as an anti-freezing wearable strain sensor. Firstly, the strain-sensing behaviors of the PHEAA-GE-Gl-LiCl organohydrogel were investigated at −40°C (Figure 8A). Similar to that at room temperature, *ΔR/R*_*0*_ of PHEAA-GE-Gl-LiCl organohydrogel based sensor at −40°C increased rapidly with the increase of strain. When the strain was 280%, the *ΔR/R*_*0*_ was 3,100%, indicating that PHEAA-GE-Gl-LiCl organohydrogel could work under a broad strain range even at −40°C. Compared with that at room temperature, the PHEAA-GE-Gl-LiCl organohydrogel displayed a higher GF value (2.59 at 0–100% strain, 7.50 at 100–172% strain, and 25.35 at 172–263% strain) at −40°C. The enhancement of GF might be attributed to the following two reasons. First, the PHEAA-GE-Gl-LiCl organohydrogel displayed a tougher network at lower temperature, resulting in restricting the movement of free ions (Figure 4D). Second, the free ions were more difficult to move in the gel network at low temperature, so the sensitivity increased more intensely during stretching. However, the deeper mechanism remained to be explored.

Same as at room temperature, the PHEAA-GE-Gl-LiCl organohydrogel-based sensor could sensitively detect both small strain and relatively large strain (5–200%) with good stability and repeatability at −40°C (Figure 8B). In addition, PHEAA-GE-Gl-LiCl organohydrogel was designed as a wearable strain sensor to detect human model motions at −40°C. As shown in Figures 8C–F, it could monitor the movement of a human model with stable and repeatable signals at −40°C, including the bending or relaxation states of various joints (e.g., finger, wrist, elbow, knee). Meanwhile, we found the relative resistance variation was positively correlated with the degree of deformation. Owing to the largest deformation, the joint motion of knee produced the highest peaks. All these results suggested the PHEAA-GE-Gl-LiCl organohydrogel-based strain sensor has a bright future in wearable devices to detect full-range human activities under a broad range of temperatures.

There were some limitations in this study. In the self-healing experiment, the operation requirement is high. First, the hydrogel should be cut with a sharp scalpel to ensure the cross section smooth and intact to the maximum extent. Second, when manually put the cut hydrogel together, we must carefully observe whether the gel section was complete contact. These operation details would influence the self-healing efficiency of the hydrogel. Further studies will optimize the effect of these experimental details on the self-healing efficiency.

## Conclusion

In summary, we have successfully developed a fully physically linked PHEAA-GE-Gl-LiCl organohydrogel *via* a facile one-pot heating-cooling-photopolymerization method. The resulting PHEAA-GE-Gl-LiCl organohydrogel exhibited superstretchability (970%), remarkable self-healing ability (the strain of self-healed organohydrogel was 263%), and a high gauge factor of 13.16. Meanwhile, on account of the presence of both glycerol and LiCl, PHEAA-GE-Gl-LiCl organohydrogel possessed excellent anti-freezing, and ultra-high strain sensitivity features, which could be designed as wearable strain sensors for strain detection in wide temperature ranges (from −40 to 25°C). The PHEAA-GE-Gl-LiCl organohydrogel based-strain sensor was able to accurately monitor and distinguish both large-scale human motions such as joint bending and subtle physiological signals such as swallowing and pronouncing. Owing to these outstanding performances, it was conceivable that the PHEAA-GE-Gl-LiCl organohydrogel would present an unpredictable prospect for use in wearable strain sensors.

## Data Availability

The original contributions presented in the study are included in the article/Supplementary Material, further inquiries can be directed to the corresponding authors.
