# Supplementary material for: Ionic Conductive Organohydrogel With Ultrastretchability, Self-Healable and Freezing-Tolerant Properties for Wearable Strain Sensor
Source: Front Chem. 2021 Oct 18;9:758844. doi: 10.3389/fchem.2021.758844 (PMC8558561; doi:10.3389/fchem.2021.758844)
Supplement: Supplementary file 1 [file Table1.docx]

**Ionic Conductive Organohydrogel With Ultrastretchability, Self-healable and Freezing-Tolerant Properties for Wearable Strain Sensor**

Feng Ji^1^, Min Jiang^1^, Qingyu Yu^2^, Xuefang Hao^3^, Yan Zhang^1^, Junqiu Zhu^1^, Shuiyuan Luo^1*^, Junjie Li^1,2,4*^

^1^ College of Chemical Engineering and Materials Science, Quanzhou Normal University, Quanzhou, 362000, China

^2^ School of Chemical Engineering and Technology, Tianjin University, Tianjin 300350, China

^3^ Nano Innovation Institute, Inner Mongolia Key Laboratory of Carbon Nanomaterials, College of Chemistry and Materials Science, Inner Mongolia University for Nationalities, Tongliao 028000, China

^4^ Frontiers Science Center for Synthetic Biology and Key Laboratory of Systems Bioengineering (Ministry of Education), Tianjin University, Tianjin 300350, China.

*Corresponding author: *Shuiyuan Luo, E-mail: syluo@qztc.edu.cn

*Junjie Li, E-mail: li41308@tju.edu.cn

1. **Figures**


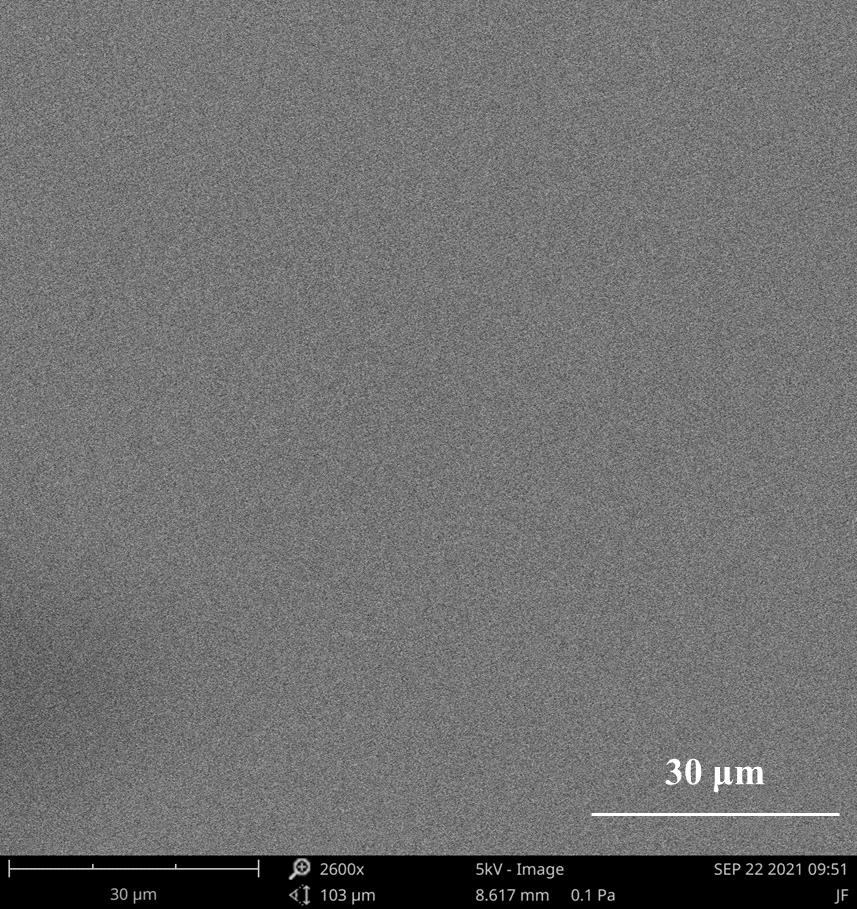


**Figure S1**. The SEM of PHEAA-GE-Gl-LiCl organohydrogel.

**
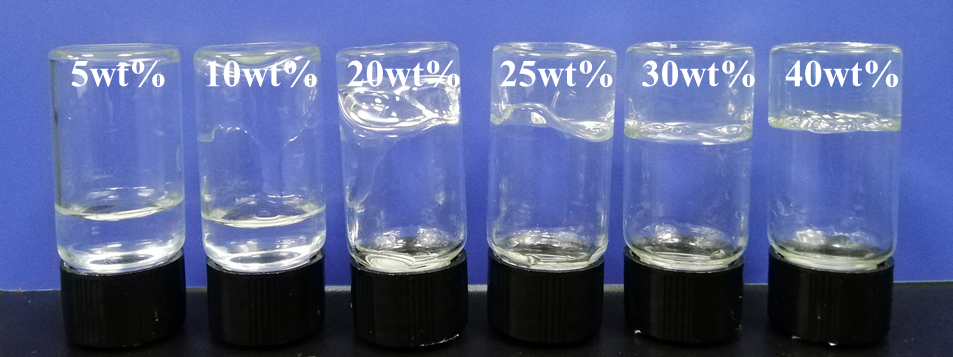
**

**Figure S2**. Photograph of PHEAA SN hydrogels prepared at different HEAA concentrations.

**
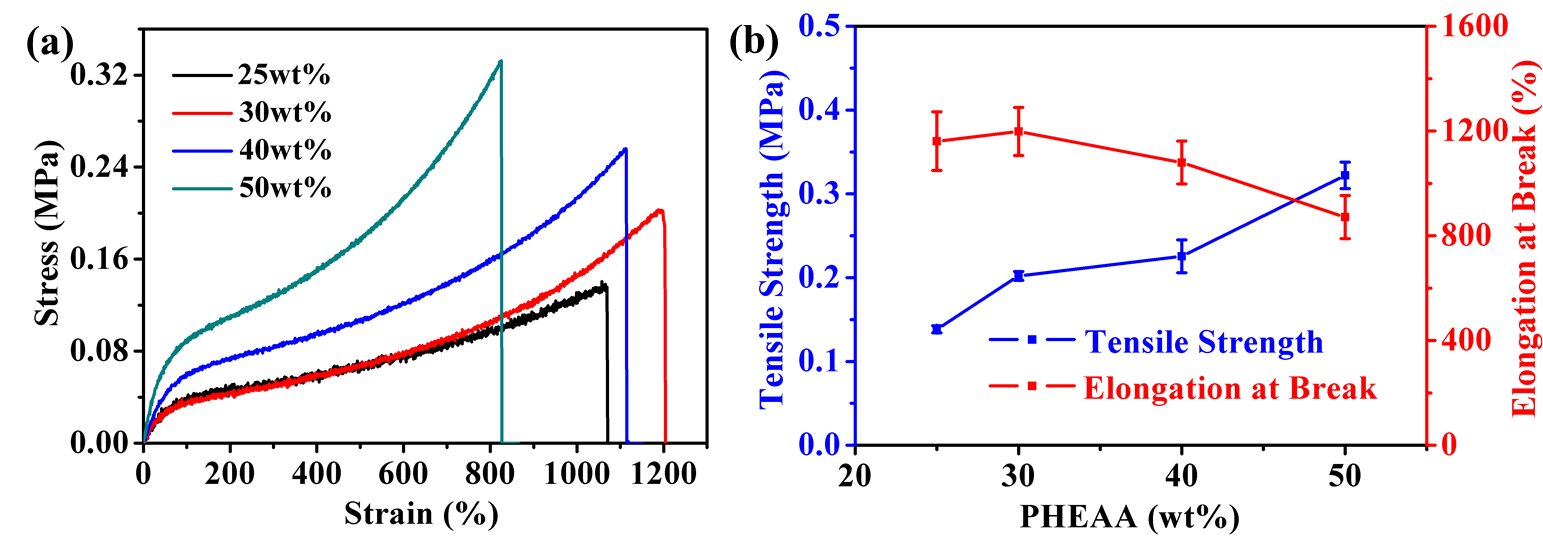
**

**Figure S3**. Mechanical properties of PHEAA SN hydrogels prepared with different concentrations. (a) The tensile stress-strain curves, (b) tensile strength and elongation at break.


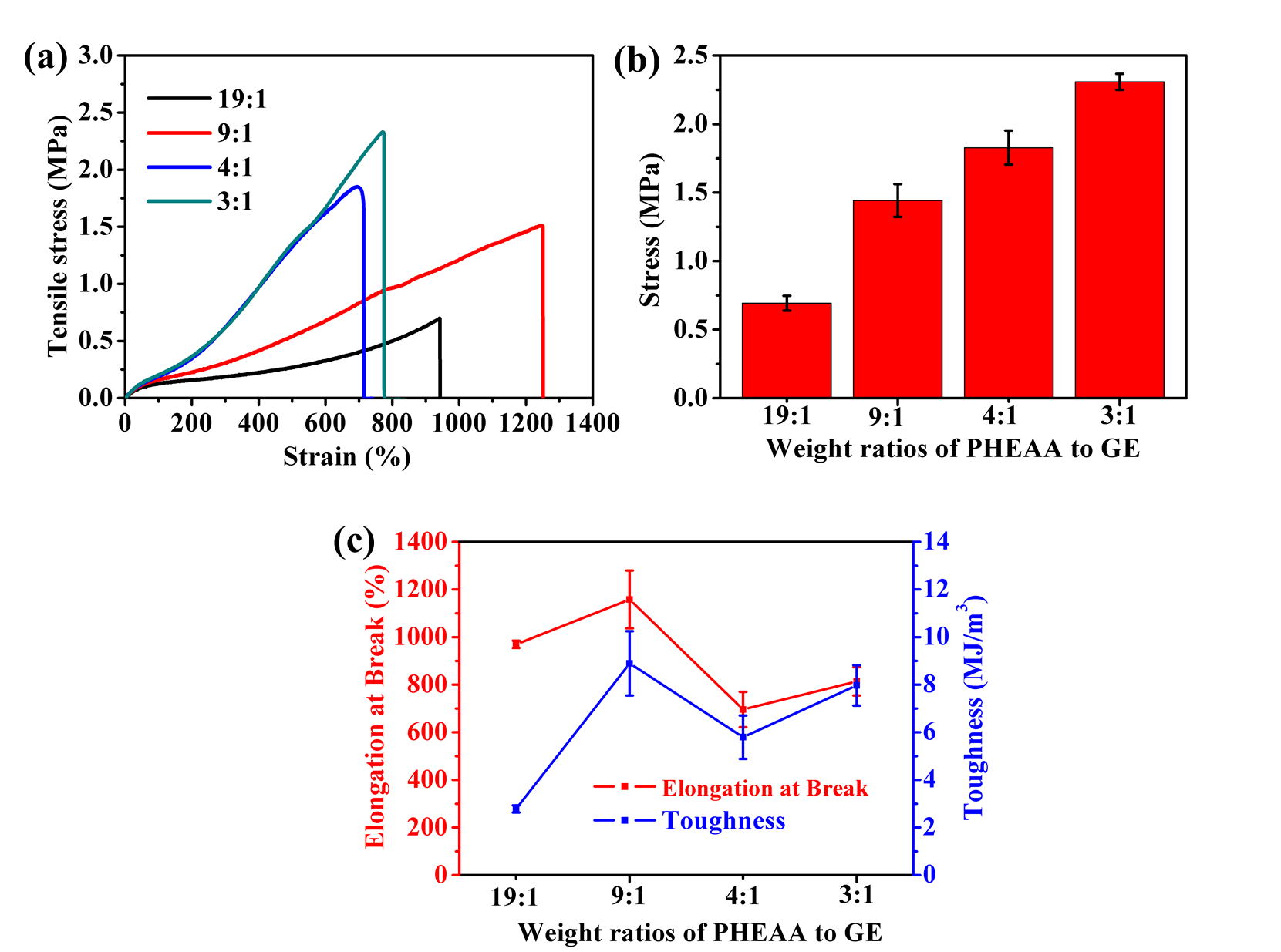


**Figure S4.** Effect of the weight ratios of PHEAA to GE on the mechanical property of PHEAA-GE DN hydrogels. (a) The tensile stress-strain curves, (b) stress, (c) elongation at break and toughness.


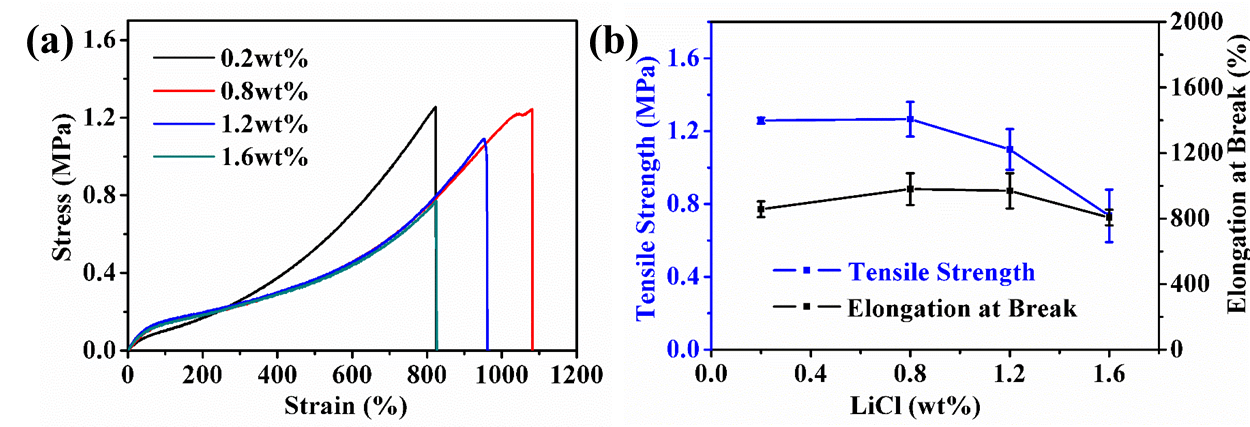


**Figure S5.** Effect of LiCl concentration on the mechanical property of PHEAA-GE-LiCl hydrogel. (a) The stress-strain curves, (b) tensile strength and elongation at break.


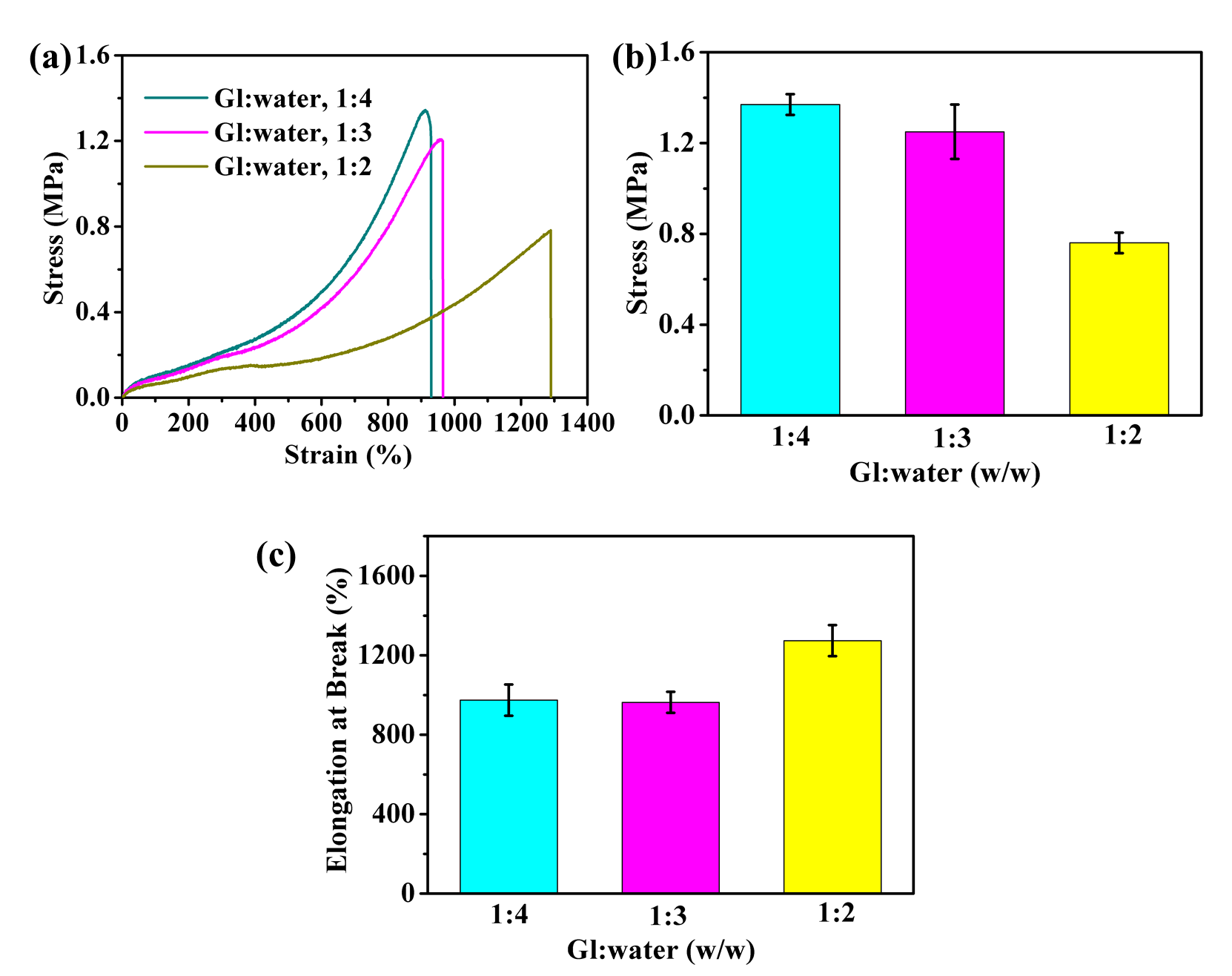


**Figure S6.** Effect of the weight ratios of Gl/water on the mechanical property of PHEAA-GE-Gl-LiCl organohydrogel. (a) The stress-strain curves, (b) stress, and (c) elongation at break.


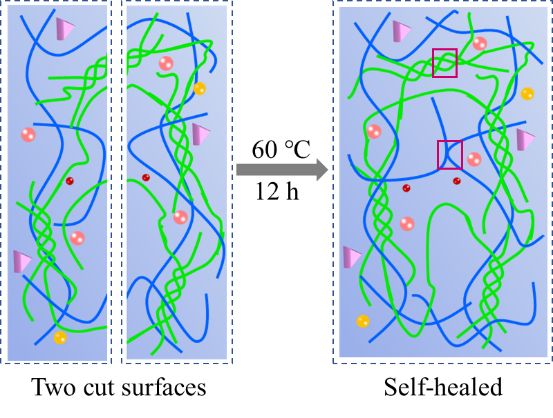


**Figure S7.** The schematic diagram of the self-healing mechanism of PHEAA-GE-Gl-LiCl organohydrogel.


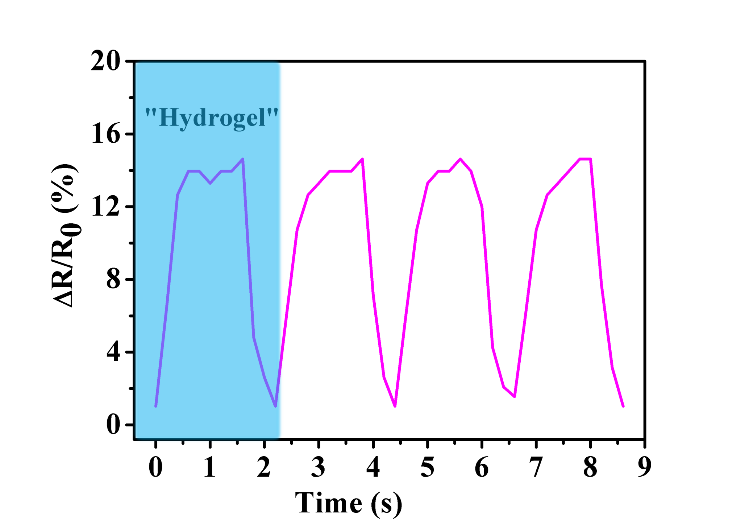


**Figure S8.** Detection of subtle motions of pronouncing word “Hydrogel”.

**2. Tables**

**Table S1.** The self-healing properties of the self-healed gel based on the reported PAAm-polysaccharide or PHEAA-polysaccharide for flexible electronic sensors.

| Composition | Self-healing | strain of the healed gel | Ref. |
| --- | --- | --- | --- |
| PHEAA-GE-Gl-LiCl | **Yes** | **263%** | **This work** |
| PAAm-GG | Yes | 120% | 1 |
| PAAm-Car-EG/Gl-KCl | Yes | 150%/170% | 2 |
| PAAm-CMCs-NaCl | Yes | 102% | 3 |
| PAAm-Car-EG/Gl-KCl | Yes | 85% | 4 |
| PAAm-Car-KCl | Yes | 83% | 5 |
| PAAm-Car-KCl | Yes | 52% | 6 |
| PAAm-GE-Cit^3-^ | — | — | 7 |
| PHEAA-Agar-[EMIM]Cl | — | — | 8 |
| PHEAA-Cs-Cit^3-^ | — | — | 9 |
| PAAm-CMC-LiCl | — | — | 10 |
| PAAm-CMC-Fe^3+^ | — | — | 11 |
| PAAm-Agar-LiCl | — | — | 12 |
| PAAm-Agar-NaCl | — | — | 13 |
| PAAm-Agar-Li_2_SO_4_ | — | — | 14 |
| PAAm-SA-NaCl | — | — | 15 |
| PAAm-XG-Fe^3+^ | — | — | 16 |

Note: “—” indicates “not available” in the references.

**Table S2.** The properties of reported anti-freezing hydrogel-based strain sensors.

| Composition | Low temperature | | Ref. |
| --- | --- | --- | --- |
|  | **Workable temperature (°C)** | **Strain in workable**  **temperature (%)** |  |
| PHEAA-GE-Gl-LiCl | **-40** | **608** | **This work** |
| PAAm-XG-Fe^3+^ | -40 | 530 | 16 |
| GE-Cit^3-^ | -30 | 400 | 17 |
| PVA-Gl-NaCl | -20 | 510 | 18 |
| PAAm-Car-Eg/Gl-KCl | -18 | 950 | 2 |
| Cellulose-Zn^2+^-Ca^2+^ | -60 | 85 | 19 |
| PAAm-PAA-Fe^3+^-NaCl | -24.7 | — | 20 |
| PAAm-PDA-CNTs-Gl | -20 | — | 21 |
| PVA-GE-NaCl | -20 | — | 22 |
| PAAm-CMCs-NaCl | -20 |  | 3 |
| PVC-CS-GO-Gl | -20 | — | 23 |
| PVA-PANi-Gl | -20 | — | 24 |
| PVA-SA-Gl-NaCl | -20 | — | 25 |
| Cellulose ionic hydrogel | -20 | — | 26 |
| PMAANa-PDMC-Gl | -20 | — | 27 |
| PVA-PEDOT-Gl | -20 | — | 28 |
| GG-borax-Gl | -18.7 | — | 29 |
| PAAm-CMC-EG-Fe^3+^ | -12 | — | 30 |

Note: “—” indicates “not available” in the references.

**Table S3.** The properties of reported conductive hydrogels for flexible electronic sensors.

| Composition | Maximal strain (%) | GF | Sensing ranges | Anti-freezing | Ref. |
| --- | --- | --- | --- | --- | --- |
| PHEAA-GE-Gl-LiCl | **970** | **13.16** | **0-500** | **Yes** | **This work** |
| PHEAA-CS-Cit^3-^ | 692 | 6.90 | 0-400 | Yes | 9 |
| P(AAm-co-AA)-CS-Fe^3+^ | 450 | 6.60 | 0-400 | No | 31 |
| PVA-Gl-NaCl | 570 | 4.01 | 0-300 | Yes | 18 |
| HP(AAm-AA)-CS-Fe^3+^ | — | 3.62 | 0-500 | No | 32 |
| PAAm-PHMA-NaCl | 2160 | 2.37 | 0-100 | No | 33 |
| PAAm-GE-Cit^3-^ | 849 | 2.04 | 0-600 | No | 7 |
| PVA-SA-Gl-NaCl | 582 | 2.00 | 0-582 | Yes | 25 |
| PAAm-PAA–Fe^3+^-NaCl | 573 | 1.96 | 0-500 | No | 20 |
| PVA-CS-GO-Gl | 700 | 1.90 | 0-300 | Yes | 23 |
| PHEAA-SA-Ca^2+^-KCl | 400 | 1.87 | 0-200 | No | 34 |
| PAAm-Agar-LiCl | 1600 | 1.80 | 0-1100 | No | 12 |
| PVA-PSBMA | 400 | 1.50 | 0-300 | No | 35 |
| PAAm-CMC-EG-Fe^3+^ | 1086 | 1.40 | 0-30 | Yes | 30 |
| PAA-Fe^3+^-GO | 630 | 1.32 | 0-500 | No | 36 |
| PVA-GE-NaCl | 715 | 0.75 | 0-700 | Yes | 22 |
| PAAm-PDA-talc | 1000 | 0.69 | 0-1000 | No | 37 |
| PAAm-Car-KCl | — | 0.63 | 0-1000 | No | 5 |
| PAAm-CMCS-NaCl | 1100 | 0.21 | 0-800 | Yes | 3 |

Note: “—” indicates “not available” in the references.

**3. References**

[S1] Liu, S., Qiu, Y., Yu, W., and Zhang, H. (2020a). Highly stretchable and self-healing strain sensor based on gellan gum hybrid hydrogel for human motion monitoring. *ACS Appl. Mater. Interfaces* 2, 1325-1334. [doi:10.1021/acsapm.9b01200](https://doi.org/10.1021/acsapm.9b01200)

[S2] Wu, J., Wu, Z., Lu, X., Han, S., Yang, B. R., Gui, X., et al. (2019a). Ultrastretchable and stable strain sensors based on antifreezing and self-healing ionic organohydrogels for human motion monitoring. *ACS Appl. Mater. Interfaces* 11, 9405-9414. [doi:10.1021/acsami.8b20267](https://doi.org/10.1021/acsami.8b20267)

[S3] Ding, H., Liang, X., Wang, Q., Wang, M., Li, Z., and Sun, G. (2020). A semi-interpenetrating network ionic composite hydrogel with low modulus, fast self-recoverability and high conductivity as flexible sensor. *Carbohydr. Polym*. 248, 116797. [doi:10.1016/j.carbpol.2020.116797](https://doi.org/10.1016/j.carbpol.2020.116797)

[S4] Wu, J., Wu, Z., Xu, H., Wu, Q., Liu, C., Yang, B. R., et al. (2019b). An intrinsically stretchable humidity sensor based on anti-drying, self-healing and transparent organohydrogels. *Materials Horizons* 6, 595-603. [doi: 10.1039/C8MH01160E](https://doi.org/10.1039/C8MH01160E)

[S5] Liu, S., and Li, L. (2017). Ultrastretchable and self-healing double-network hydrogel for 3D printing and strain sensor. *ACS Appl. Mater. Interfaces* 9, 26429-26437. [doi:10.1021/acsami.7b07445](https://doi.org/10.1021/acsami.7b07445)

[S6] Wu, J., Han, S., Yang, T., Li, Z., Wu, Z., Gui, X., et al. (2018). Highly stretchable and transparent thermistor based on self-healing double network hydrogel. *ACS Appl. Mater. Interfaces* 10, 19097-19105. [doi:10.1021/acsami.8b03524](https://doi.org/10.1021/acsami.8b03524)

[S7] Sun, X., Yao, F., Wang, C., Qin, Z., Zhang, H., Yu, Q., et al. (2020a). Ionically conductive hydrogel with fast self‐recovery and low residual strain as strain and pressure sensors. *Macromol. Rapid Commun*. 41, 2000185. [doi: 10.1002/marc.202000185](https://doi.org/10.1002/marc.202000185)

[S8] Sun, J., Lu, G., Zhou, J., Yuan, Y., Zhu, X., and Nie, J. (2020b). Robust physically linked double-network ionogel as a flexible bimodal sensor. *ACS Appl. Mater. Interfaces* 12, 14272-14279. [doi:10.1021/acsami.0c01472](https://doi.org/10.1021/acsami.0c01472)

[S9] Yang. Y., Yang. Y., Cao. Y., Wang. X., Chen. Y., Liu. H., et al. (2021). Anti-freezing, resilient and tough hydrogels for sensitive and large-range strain and pressure sensors, *Chem. Eng. J.* 403 126431. [https://doi.org/10.1016/j.cej.2020.126431](https://doi.org/10.1016/j.cej.2020.126431).

[S10] Zhu, T., Cheng, Y., Cao, C., Mao, J., Li, L., Huang, J., et al. (2020). A semi-interpenetrating network ionic hydrogel for strain sensing with high sensitivity, large strain range, and stable cycle performance. *Chem. Eng. J.* 385, 123912. doi:10.1016/j.cej.2020.126431

[S11] Zhang, H., Wu, X., Qin, Z., Sun, X., Zhang, H., Yu, Q., et al. (2020). Dual physically cross-linked carboxymethyl cellulose-based hydrogel with high stretchability and toughness as sensitive strain sensors. *Cellulose* 27, 9975-9989. [doi: 10.1007/s10570-020-03463-5](https://doi.org/10.1007/s10570-020-03463-5)

[S12] Yang, B., and Yuan, W. (2019). Highly stretchable and transparent double-network hydrogel ionic conductors as flexible thermal-mechanical dual sensors and electroluminescent devices. *ACS Appl. Mater. Interfaces* 11, 16765-16775. [doi:10.1021/acsami.9b01989](https://doi.org/10.1021/acsami.9b01989)

[S13] Hou, W., Sheng, N., Zhang, X., Luan, Z., Qi, P., Lin, M., et al. (2019). Design of injectable agar/NaCl/polyacrylamide ionic hydrogels for high performance strain sensors. *Carbohydr. Polym*. 211, 322-328. [doi: 10.1016/j.carbpol.2019.01.094](https://doi.org/10.1016/j.carbpol.2019.01.094)

[S14] Lin, T., Shi, M., Huang, F., Peng, J., Bai, Q., Li, J., et al. (2018). One-pot synthesis of a double-network hydrogel electrolyte with extraordinarily excellent mechanical properties for a highly compressible and bendable flexible supercapacitor. *ACS Appl. Mater. Interfaces* 10, 29684-29693. [doi: 10.1021/acsami.8b11377](https://doi.org/10.1021/acsami.8b11377)

[S15]Zhang, X., Sheng, N., Wang, L., Tan, Y., Liu, C., Xia, Y., et al. (2019a). Supramolecular nanofibrillar hydrogels as highly stretchable, elastic and sensitive ionic sensors. *Materials Horizons* 6, 326-333. [doi:10.1039/C8MH01188E](https://doi.org/10.1039/C8MH01188E)

[S16] Yu, Q., Qin, Z., Ji, F., Chen, S., Luo, S., Yao, M., et al. (2021). Low-temperature tolerant strain sensors based on triple crosslinked organohydrogels with ultrastretchability. *Chem. Eng. J*. 404, 126559. [doi: 10.1016/j.cej.2020.126559](https://doi.org/10.1016/j.cej.2020.126559)

[S17] Qin, Z., Sun, X., Zhang, H., Yu, Q., Wang, X., He, S., et al. (2020). A transparent, ultrastretchable and fully recyclable gelatin organohydrogel based electronic sensor with broad operating temperature. *J. Mater. Chem. A* 8, 4447-4456. doi: [10.1039/C9TA13196E](https://doi.org/10.1039/C9TA13196E)

[S18] Pan, S., Xia, M., Li, H., Jiang, X., He, P., Sun, Z., et al. (2020). Transparent, high-strength, stretchable, sensitive and anti-freezing poly (vinyl alcohol) ionic hydrogel strain sensors for human motion monitoring. *J. Mater. Chem. C* 8, 2827-2837. [doi: 10.1039/C9TC06338B](https://doi.org/10.1039/C9TC06338B)

[S19] Zhang, X. F., Ma, X., Hou, T., Guo, K., Yin, J., Wang, Z., et al. (2019b). Inorganic salts induce thermally reversible and anti‐freezing cellulose hydrogels. *Angew. Chem. Int. Ed*. 58, 7366-7370. [doi: 10.1002/anie.201902578](https://doi.org/10.1002/anie.201902578)

[S20] Li, S., Pan, H., Wang, Y., and Sun, J. (2020). Polyelectrolyte complex-based self-healing, fatigue-resistant and anti-freezing hydrogels as highly sensitive ionic skins. *J. Mater. Chem. A* 8, 3667-3675. [doi: 10.1039/C9TA13213A](https://doi.org/10.1039/C9TA13213A)

[S21] Han, L., Liu, K., Wang, M., Wang, K., Fang, L., Chen, H., et al. (2018). Mussel‐inspired adhesive and conductive hydrogel with long‐lasting moisture and extreme temperature tolerance. *Adv. Funct. Mater.* 28, 1704195. [doi: 10.1002/adfm.201704195](https://doi.org/10.1002/adfm.201704195)

[S22] Chen, H., Ren, X., and Gao, G. (2019). Skin-Inspired gels with toughness, antifreezing, conductivity, and remoldability. *ACS Appl. Mater. Interfaces 11*, 28336-28344. [doi: 10.1021/acsami.9b11032](https://doi.org/10.1021/acsami.9b11032)

[S23] Yang, T., Wang, M., Jia, F., Ren, X., and Gao, G. (2020). Thermo-responsive shape memory sensors based on tough, remolding and anti-freezing hydrogels. *J. Mater. Chem. C* 8, 2326-2335. [doi: 10.1039/C9TC05804D](https://doi.org/10.1039/C9TC05804D)

[S24] Hu, C., Zhang, Y., Wang, X., Xing, L., Shi, L., and Ran, R. (2018). Stable, strain-sensitive conductive hydrogel with antifreezing capability, remoldability, and reusability. *ACS Appl. Mater. Interfaces* 10, 44000-44010. [doi: 10.1021/acsami.8b15287](https://doi.org/10.1021/acsami.8b15287)

[S25] Chen, H., Gao, Y., Ren, X., and Gao, G. (2020). Alginate fiber toughened gels similar to skin intelligence as ionic sensors. *Carbohydr. Polym.* 235, 116018. [doi: 10.1016/j.carbpol.2020.116018](https://doi.org/10.1016/j.carbpol.2020.116018)

[S26] Tong, R., Chen, G., Pan, D., Qi, H., Li, R. A., Tian, J., et al. (2019). Highly stretchable and compressible cellulose ionic hydrogels for flexible strain sensors. *Biomacromolecules* 20, 2096-2104. [doi:10.1021/acs.biomac.9b00322](https://doi.org/10.1021/acs.biomac.9b00322)

[S27] Yang, Y., Guan, L., Li, X., Gao, Z., Ren, X., and Gao, G. (2018). Conductive organohydrogels with ultrastretchability, antifreezing, self-healing, and adhesive properties for motion detection and signal transmission. *ACS Appl. Mater. Interfaces* 11, 3428-3437. [doi:10.1021/acsami.8b17440](https://doi.org/10.1021/acsami.8b17440)

[S28] Peng, Y., Yan, B., Li, Y., Lan, J., Shi, L., and Ran, R. (2020). Antifreeze and moisturizing high conductivity PEDOT/PVA hydrogels for wearable motion sensor. *J. Mater. Sci* 55, 1280-1291. d[oi:10.1007/s10853-019-04101-7](https://doi.org/10.1007/s10853-019-04101-7)

[S29] Pan, X., Wang, Q., Ning, D., Dai, L., Liu, K., Ni, Y., et al. (2018). Ultraflexible self-healing guar gum-glycerol hydrogel with injectable, antifreeze, and strain-sensitive properties. *ACS Biomater. Sci. Eng* 4, 3397-3404. [doi:10.1021/acsbiomaterials.8b00657](https://doi.org/10.1021/acsbiomaterials.8b00657)

[S30] Cheng, Y., Ren, X., Gao, G., and Duan, L. (2019). High strength, anti-freezing and strain sensing carboxymethyl cellulose-based organohydrogel. *Carbohydr. Polym.* 223, 115051. [doi: 10.1016/j.carbpol.2019.115051](https://doi.org/10.1016/j.carbpol.2019.115051)

[S31] Liu, H., Wang, X., Cao, Y., Yang, Y., Yang, Y., Gao, Y., et al. (2020b). Freezing-tolerant, highly sensitive strain and pressure sensors assembled from ionic conductive hydrogels with dynamic cross-links. *ACS Appl. Mater. Interfaces* 12, 25334-25344. [doi: 10.1016/j.carbpol.2019.115051](https://doi.org/10.1021/acsami.0c06067)

[S32] Xu, J., Jin, R., Ren, X., and Gao, G. (2019). Cartilage-inspired hydrogel strain sensors with ultrahigh toughness, good self-recovery and stable anti-swelling properties. *J. Mater. Chem. A* 7*,* 25441-25448. [doi: 10.1039/C9TA09170J](https://doi.org/10.1039/C9TA09170J)

[S33] Zhang, Q., Liu, X., Ren, X., Jia, F., Duan, L., and Gao, G. (2019c). Nucleotide-regulated tough and rapidly self-recoverable hydrogels for highly sensitive and durable pressure and strain sensors. *Chem. Mater.* 31, 5881-5889. [doi:10.1021/acs.chemmater.9b02039](https://doi.org/10.1021/acs.chemmater.9b02039)

[S34] Song, J., Chen, S., Sun, L., Guo, Y., Zhang, L., Wang, S., et al. (2020). Mechanically and electronically robust transparent organohydrogel fibers. *Adv. Mater.* 32, 1906994. [doi: 10.1002/adma.201906994](https://doi.org/10.1002/adma.201906994)

[S35] Wang, Z., Chen, J., Wang, L., Gao, G., Zhou, Y., Wang, R., et al. (2019). Flexible and wearable strain sensors based on tough and self-adhesive ion conducting hydrogels. *J. Mater. Chem. B* 7, 24-29. [doi:10.1039/c8tb02629g](https://doi.org/10.1039/c8tb02629g)

[S36] Jing, X., Mi, H. Y., Peng, X. F., and Turng, L. S. (2018a). Biocompatible, self-healing, highly stretchable polyacrylic acid/reduced graphene oxide nanocomposite hydrogel sensors via mussel-inspired chemistry. *Carbon* 136, 63-72. [doi:10.1016/j.carbon.2018.04.065](%5bS36%5d%20X.%20Jing,%20H.-Y.%20Mi,%20X.-F.%20Peng,%20L.-S.%20Turng,%20Biocompatible,%20self-healing,%20highly%20stretchable%20polyacrylic%20acid/reduced%20graphene%20oxide%20nanocomposite%20hydrogel%20sensors%20via%20mussel-inspired%20chemistry,%20Carbon%20136%20(2018)%2063-72.%20https:/doi.org/10.1016/j.carbon.2018.04.065.)

[S37] Jing, X., Mi, H. Y., Lin, Y. J., Enriquez, E., Peng, X. F., and Turng, L. S. (2018b). Highly stretchable and biocompatible strain sensors based on mussel-inspired super-adhesive self-healing hydrogels for human motion monitoring. *ACS Appl. Mater. Interfaces* 10, 20897-20909. [doi:10.1021/acsami.8b06475](https://doi.org/10.1021/acsami.8b06475)
